# GWAS and Meta-QTL Analysis of Yield-Related Ear Traits in Maize

**DOI:** 10.3390/plants12223806

**Published:** 2023-11-08

**Authors:** Fu Qian, Jianguo Jing, Zhanqin Zhang, Shubin Chen, Zhiqin Sang, Weihua Li

**Affiliations:** 1 Xinjiang Academy of Agricultural and Reclamation Science, Shihezi 832000, China; 20202012013@stu.shzu.edu.cn (F.Q.); zzq3000qwe@163.com (Z.Z.); btcorn@163.com (S.C.); 2 The Key Laboratory of Oasis Eco-Agriculture, College of Agriculture, Shihezi University, Shihezi 832003, China; jingjianguo2023@163.com

**Keywords:** maize, ear traits, yield, GWAS, candidate genes, meta-QTL

## Abstract

Maize ear traits are an important component of yield, and the genetic basis of ear traits facilitates further yield improvement. In this study, a panel of 580 maize inbred lines were used as the study material, eight ear-related traits were measured through three years of planting, and whole genome sequencing was performed using the maize 40 K breeding chip based on genotyping by targeted sequencing (GBTS) technology. Five models were used to conduct a genome-wide association study (GWAS) on best linear unbiased estimate (BLUE) of ear traits to find the best model. The FarmCPU (Fixed and random model Circulating Probability Unification) model was the best model for this study; a total of 104 significant single nucleotide polymorphisms (SNPs) were detected, and 10 co-location SNPs were detected simultaneously in more than two environments. Through gene function annotation and prediction, a total of nine genes were identified as potentially associated with ear traits. Moreover, a total of 760 quantitative trait loci (QTL) associated with yield-related traits reported in 37 different articles were collected. Using the collected 760 QTL for meta-QTL analysis, a total of 41 MQTL (meta-QTL) associated with yield-related traits were identified, and 19 MQTL detected yield-related ear trait functional genes and candidate genes that have been reported in maize. Five significant SNPs detected by GWAS were located within these MQTL intervals, and another three significant SNPs were close to MQTL (less than 1 Mb). The results provide a theoretical reference for the analysis of the genetic basis of ear-related traits and the improvement of maize yield.

## 1. Introduction

Maize (*Zea mays* L.) is one of the three major food crops and plays an important role in global food security. However, with rapid economic development and an increase in population, the area of land cultivated has decreased, leading to severe food security challenges. As a result, high yield has become a primary goal in maize breeding [1]. Genetic breeding is one of the main ways to improve maize yield. Maize yield is a quantitative trait controlled by multiple genes, and is the result of the comprehensive action of multiple agronomic traits. The genetic structure is extremely complex and easily interacts with the environment. Maize yield was affected by reproductive stage, plant type and ear traits. The ear diameter, ear row number, kernel number per row, and hundred kernel weight had a great effect on yield [2]. With the continuous analysis and research on the various factors affecting maize yield, it has been found that yield is mainly related to maize ear traits. The heritability of yield-related ear traits possess higher than that grain yield, and ear traits have a great impact on improving grain yield [3]. Therefore, dissecting the genetic and molecular mechanisms of ear traits with the aim of improving maize yield is an important problem to be solved in current agricultural production.

GWAS is a method to screen the high-density molecular markers in the entire genome of a population, and analyze the associations between the molecular marker data and phenotypic traits. The method is an efficient research tool for analyzing the inheritance of quantitative traits [4]. With the rapid development of molecular marker technology, the positioning accuracy and detection accuracy of GWAS have also been greatly improved, and it has become a common method to study the genetic mechanism of natural phenotypic variation in plants [5]. At present, there are corresponding studies on quantitative trait mapping and association analysis of maize ear traits. One single-locus method and six multi-locus methods of GWAS were used to identify significant quantitative trait nucleotides for kernel number per row in an association panel that included 639 maize inbred lines [6]. Zhang et al. used an association panel comprising 362 inbred lines and a recombinant inbred line population derived from X178 × 9782 to identify candidate genes for nine yield traits [7]. Brown et al. identified 36 QTL and 261 significant SNPs for kernel number per row in a nested association mapping population through joint linkage and GWAS [8]. Yang et al. detected a total of 116 significant loci based on 126 maize inbred lines, 42 of which were related to the ear row number, and 37 were related to the kernel number per row, and predicted some possible functional genes, such as *ts6*, *pin4*, *Zm00001d038022* and *Zm00001d041584* [9]. Zhang et al. combined GWAS and QTL analysis for dissecting the genetic architecture of kernel test weight in maize; a total of 18 significant volumetric-related SNPs were identified using GWAS, *Zm00001d044075*, *Zm00001d044086*, and *Zm00001d044081* were further identified by candidate gene association study for volume weight [10].

Although many QTL and SNPs for ear traits have been reported, there are differences in experimental environments, mapping populations, population types, trait selection, statistical methods, etc., among different studies, making it difficult to detect all QTL controlling traits in a single QTL mapping experiment [11]. Due to the limitation of marker density, the genetic distance of the QTL confidence interval located is relatively large, and the effectiveness of QTL is relatively low. Meta analysis can optimize the confidence interval of QTL using mathematical models based on the integration of different research positioning QTL, thereby improving the accuracy and effectiveness of QTL positioning [12]. Meta-analysis has been used for QTL integration, consensus map construction, candidate gene fine mapping, and discovery of major traits in maize [13,14].

Although there have been previous studies, there are still relatively few studies on the combined analysis using GWAS and meta-QTL for ear traits related to maize yield. It is necessary to use multiple methods to conduct association analyses of different populations in different environments to find stable association loci and excellent alleles for the high-yield breeding of maize. In this study, a natural population composed of 580 maize inbred lines from different sources was used to perform whole genome sequencing using genotyping by GBTS [15]. GWAS was performed on yield-related ear traits, such as ear row number (ERN), kernel number per row (KNR), ear length (EL), ear diameter (ED), cob diameter (CD), ear weight (EW), hundred kernel weight (HKW) and volume weight (VW), to analyze the genetic basis of maize yield-related traits, identify SNPs loci and candidate genes significantly associated with ears traits, and, additionally, to integrate QTL of multiple ear traits, use meta-analysis methods to determine “consistent” QTL (Meta-QTL) related to maize ear traits, search for candidate genes, and provide a basis for precise localization and cloning of important genes in maize ear traits, providing the theoretical basis and technical support for improving maize yield through molecular marker-assisted selection breeding.

## 2. Results

### 2.1. Phenotypic Analysis of Ear Traits

**Figure 1:**
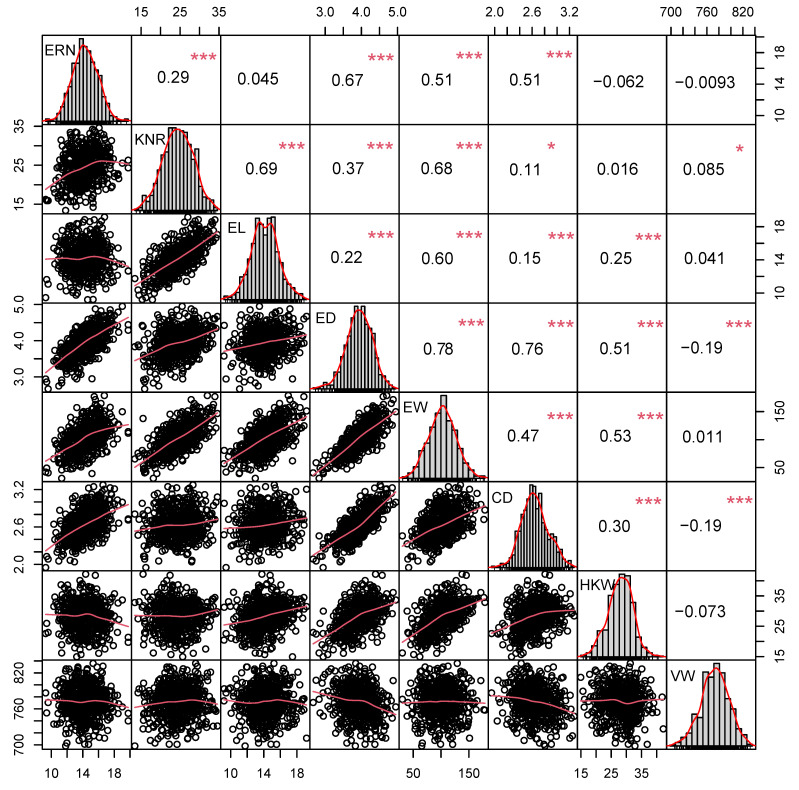
Correlation analysis of BLUE value of ear traits. ERN, ear row number; KNR, kernel number per row; EL, ear length; ED, ear diameter; CD, cob diameter; EW, ear weight; HKW, hundred kernel weight; VW, volume weight. * and *** indicates the significant levels at *p* < 0.05 and 0.001, respectively.

The basic statistical analysis showed that all traits extensive phenotypic continuous variation, with coefficients of variation ranging from 3.24% to 26.71% (Table S1), and eight ear traits obeyed a normal distribution (Figure 1), which indicated that the ear traits are typical quantitative traits, and their inheritance is mainly controlled by polygenes. The *H*^2^ (heritability) of the eight ear traits were relatively high, ranging from 70.25% to 89.04% (Table 1), indicating that a large portion of phenotypic variance for ear traits could be attributed to genotypic effects.

The analysis of variance (Table 1) showed that the genotype effect, environment (year) effect, and genotype–environment interaction effect of ERN, KNR, EL, ED, EW, CD and HKW all reached extremely significant levels. This showed that there was significant genetic variation in these seven ear traits and they were strongly affected by genotype, environment, and genotype–environment interactions. The interaction effect of the VW genotype and environment was not significant, indicating that VW was greatly affected by both genotype and environment, but not affected by the interaction between genotype and environment.

Correlation analysis showed that EW significantly and positively correlated with ERN, KNR, ED, CD, EL and HKW, but not with VW (Figure 1, Table S2). There were extremely significant positive correlations among multiple ear traits, indicating that the maize yield-related ear traits interacted, influenced and cooperated with each other, thereby regulating and affecting the yield of maize. Based on the analysis of various phenotypic traits, eight yield-related ear traits were suitable for GWAS.

### 2.2. Population Structure and Kinship Analysis

**Figure 2:**
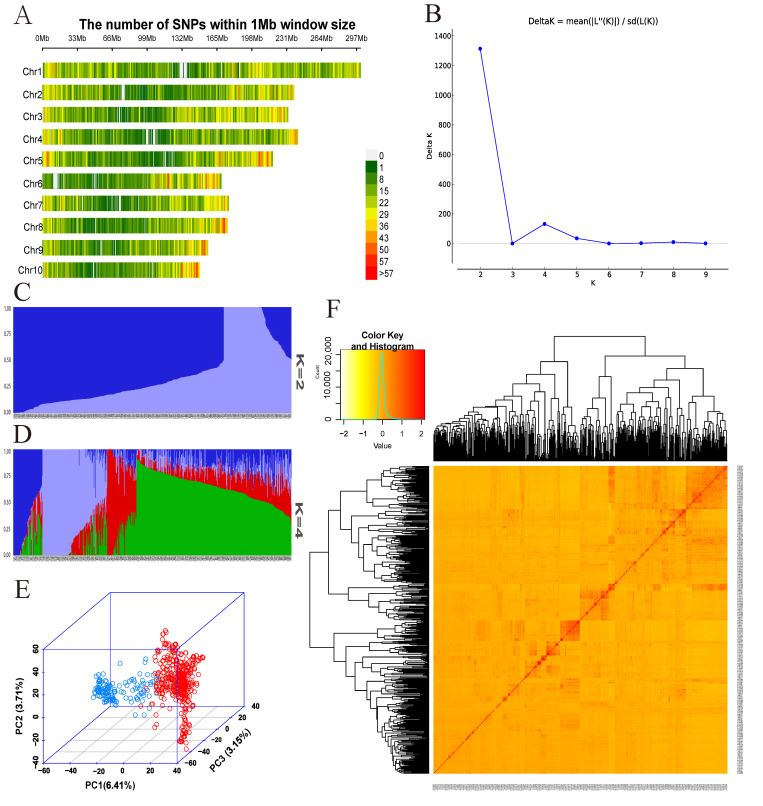
Genetic background and population structure analysis of 580 maize inbred lines. (**A**) Density distribution of SNPs on chromosome. (**B**) ΔK-value of 580 inbred lines based on 31,826 SNPs. (**C**) The Bayes cluster analysis of 580 maize inbred lines when K = 2. (**D**) The Bayes cluster analysis of 580 maize inbred lines when K = 4. (**E**) Principal component analysis of 580 maize inbred lines. The different groups represented by different colors, and scattered points with the same color are basically clustered together. (**F**) Kinship heatmap.

The 31,826 high-quality SNPs remaining after quality control were distributed on 10 chromosomes, and the number of SNPs on individual chromosomes ranged from 2308 on chromosome 10 to 4767 on chromosome 1. The number of SNPs per 1 Mb window size across the whole genome (Figure 2A) showed that the distribution density of SNPs at both ends of each chromosome was relatively high, while the distribution density of SNPs in the middle of the chromosome was relatively low.

The population structure analysis found that when K = 2, the ΔK value had the largest peak (Figure 2B); therefore, the 580 maize inbred lines could be divided into two groups (Figure 2C); one group was for 141 materials and one group was for 439 materials. When K = 4, an inflection point appeared, and a second peak appeared in the ΔK value (Figure 2B), dividing the material into four subgroups (Figure 2D). Principal component analysis (PCA) showed that 580 materials could be divided into two groups (Figure 2E), which was basically the same as the results of population structure analysis.

According to the heat map of kinship relationship (Figure 2F), we can know the distance of the kinship relationship among the materials in the group; the darker the color, the higher the kinship coefficient, the higher the similarity between materials, and the more likely they belonged to the same subgroup. The kinship coefficient was mainly concentrated between −0.3 and 0.5, the genetic relationship structure was relatively simple, and most of the inbred lines in the population might be unrelated or distant.

### 2.3. GWAS Analysis

In order to find an optimal model for GWAS of ear traits related to maize yield, five common models for association analysis, including the general linear model (GLM), mixed linear model (MLM), multiple loci mixed model (MLMM), Bayesian-information and linkage-disequilibrium iteratively nested keyway (BLINK), and FarmCPU model, were used for GWAS of BLUE values of eight ear traits. The results show (Figure S1) that the GLM model had weak ability to control the false positive rate, and the false positive rate of association analysis results was high. The results of GWAS of MLM and MLMM model were relatively consistent. Although MLM and MLMM models reduced the false positive results compared with the GLM model, there were some false negative results. The BLINK and FarmCPU models could well control the false positive and false negative results of association analysis, but the FarmCPU model has better ability to control the false positive and false negative results and had a higher statistical effect. Therefore, the FarmCPU model was selected as the best association model for GWAS.

**Figure 3:**
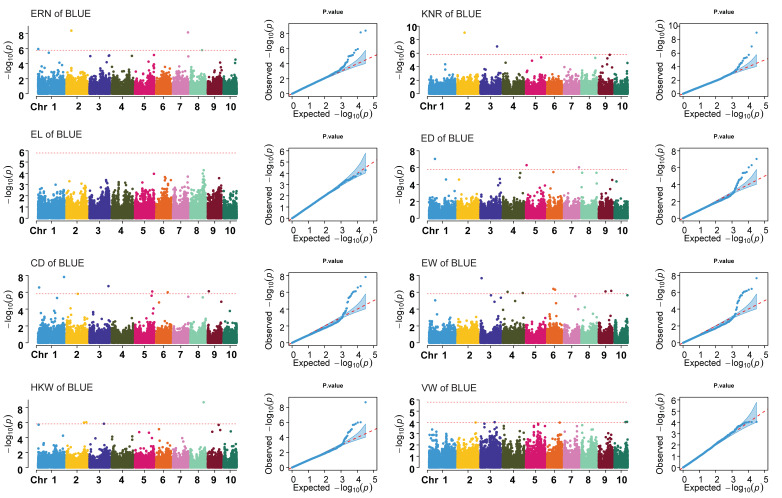
Manhattan plot and QQ plot of BLUE value of ear traits. ERN, ear row number; KNR, kernel number per row; EL, ear length; ED, ear diameter; CD, cob diameter; EW, ear weight; HKW, hundred kernel weight; VW, volume weight; BLUE, best linear unbiased estimate. The red dashed line is the threshold line.

GWAS was performed on eight yield-related ear traits using the best association model (FarmCPU). PCA and kinship were used as covariates in the association analysis to control false positives. Manhattan and QQ plots show that false positives for each trait were well controlled. (Figure 3 and Figure S2).

In the four environments (three years and BLUE value), a total of 104 SNPs significantly associated with eight ear traits were detected, including 21 for the ERN, 6 for the KNR, 11 for the CD, 7 for the EL, 22 for the EW, 21 for the HKW, and 7 for the VW (Table S3). To ensure the reliability of the screened SNPs, SNPs that were repeatedly detected in at least two environments were selected for subsequent analysis. Among the 104 SNPs detected in eight traits, ERN co-localized 2 SNPs on chromosomes 1 and 5; CD co-localized 1 SNPs on chromosome 5; EW co-localized 2 SNPs on chromosomes 6 and 9; and VW co-localized 1 SNPs on chromosome 3. There were three co-localization SNPs between ERN and ED, which were distributed on chromosomes 3 and 7; there was one co-localization SNP between EW and HKW, which was distributed on chromosome 9. No co-localized SNPs were detected for KNR, EL and ED (Table 2). The significant SNPs were co-localized between the two traits, suggesting that the SNPs may be associated with the genes controlling the two traits or a pleiotropic gene.

### 2.4. Candidate Genes Screening and Function Analysis

According to the co-localized SNPs combined with the linkage disequilibrium (LD) decay distance of 440 kb (*r*^2^ = 0.1) in this population, candidate genes were searched in the B73_RefGen_v4 reference genome, and the 440 kb interval upstream and downstream of the SNPs was used as a candidate region. Thirty-six genes were detected in the ERN candidate region, 23 genes were detected in the ED candidate region, 33 genes were detected in the EW candidate region, and 5 genes were detected in the VW candidate region. Additionally, 43 genes were detected in the ERN and ED co-localized candidate regions, but no genes were detected in the EW and HKW co-localized candidate regions (Table S4).

To determine whether these candidate genes function in a tissue specifically expressed manner, the RNA-seq data of endosperm, embryo and seeds after different days of pollination, V18 immature cob, R1 pre pollination cob, R1 anthers, R1 silk [16], ear primordium 2 mm–4 mm and 6 mm–8 mm [17], the relevant stage and tissue for ear development, were downloaded from the public database to filter genes that play roles in the development of ear. After filtering, 103 genes were identified as being expressed in ear tissues out of 140 genes in the candidate region, and some genes were highly expressed in relevant stages and tissues of ear development (Figure S6, Table S5).

Based on the genes’ functional annotation, nine possible candidate genes were identified that mainly encode various proteins and transcripts related to growth and development factors (Table 3). According to the tissue-specific expression heatmap of candidate genes (Figure S6), except for *Zm00001d013172* and *Zm00001d047736*, which are basically not expressed in ear development-related sites and tissues, *Zm00001d044069* is not expressed in other sites and tissues, but is expressed in 2 mm–4 mm and 6 mm–8 mm ear primordium, and the other six candidate genes have relatively high gene expression in the parts and tissues of ear development-related stages.

### 2.5. QTL Distribution on Maize Genome for Ear Traits

This study collected QTL for seven ear-related traits (ERN, KNR, EL, ED, CD, EW, and HKW) from 39 related articles, resulting in a collection of 760 ear-related QTL that met the predefined criteria. These were unevenly distributed on each chromosome, ranging from 44 to 123 per chromosome. Chromosome 6 had the lowest number of QTL and chromosome 1 had the highest number of QTL. Among them, there were a large number of QTL related to EL, ERN and HKW, and the minimum number of QTL related to CD. The number of QTL related to the ERN, KNR, EL, ED, CD, EW and HKW were 201, 77, 111, 85, 33, 61 and 192, respectively (Figure S7).

### 2.6. Meta-QTL Analysis

**Figure 4:**
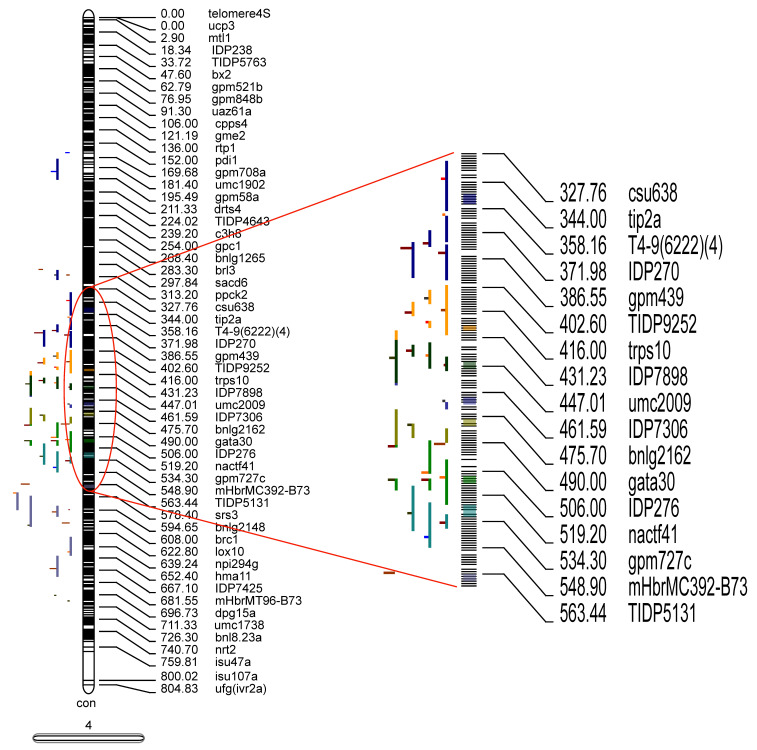
Projection and distribution of QTL and MQTL (Meta QTL) identified for ear traits on chromosome 4. Bars on the left side of the chromosome correspond to QTL related to ear traits, black bars within chromosomes represent marker density, colored segments within the chromosome represent MQTL, and on the right side of the chromosome are molecular markers and genetic distances (cM).

Maize yield QTL collected in this study were mapped by different research groups using diverse molecular markers and genetic maps. To extract valuable and sufficient information from the collected QTL for the following research, the collected QTL were projected onto a published reference map IBM2 2008 Neighbors. A total of 380 QTL (46.58% of the total QTL) collected from the literature were projected on to this newly developed highly dense reference map (Figure 4 and Figure S8), and the remaining QTL could not be projected onto the reference map. The reason for the lower percentage of QTL projection might be the lack of common markers across the original and the reference map, or the QTL showed low phenotypic variance explained (PVE) causing a large confidence interval (CI) [18].

In total, 41 MQTL related to ear traits were identified based on models with the lowest AIC (Akaike information content) values by means of meta-analysis. Each MQTL contained 3 to 12 initial QTL. Forty-one MQTL were distributed unevenly in 10 maize chromosomes: three MQTL on chromosome 1; two MQTL on each of chromosomes 2, 3, 7 and 8; seven MQTL on each of chromosomes 4, 5 and 9; four MQTL on chromosome 6; and five MQTL on chromosome 10. The CI (95%) of the detected meta-QTL varied from 0.38 to 7.87 cM with an average of 3.97 cM (Table S6). Two MQTLs (MQTL6 and MQTL7) had some overlapping areas.

By comparing the positions of the genetic markers at both ends of the MQTL on the B73 genome (AGPv4), a total of 1691 candidate genes were identified in these MQTL regions (Table S7); a maximum of 113 candidate genes were identified in MQTL 14, while a minimum of 2 candidate genes were identified in MQTL 22. Twenty-nine yield-related ear trait functional genes and candidate genes reported in maize were detected in 19 MQTL (Table S6), indicating that using meta-analysis to explore genes related to maize yield traits is efficient and feasible.

**Figure 5:**
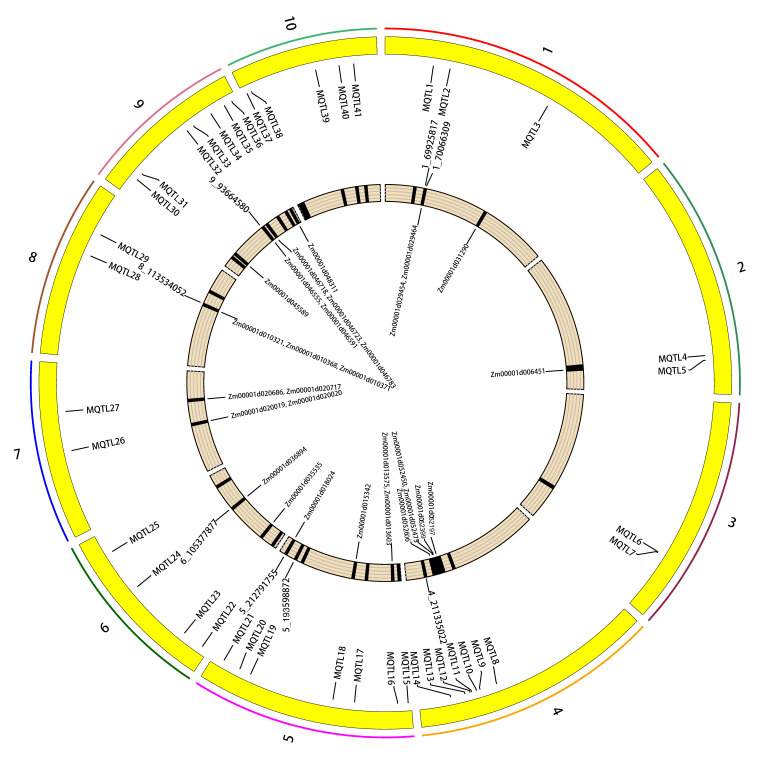
Circos plot for the distribution of MQTL (Meta QTL) and significant SNPs of GWAS studies in maize. Colored bars showing the ten maize chromosomes, yellow trajectory represents the physical positions (bp) of MQTL and SNPs on the chromosome, black area represents the physical intervals of MQTL on the chromosome.

The 104 significant SNPs of ear traits detected by GWAS in this study were compared with the physical coordinates of the 41 MQTL. The results showed that five of the detected SNPs were located within the intervals of these meta-QTL, such as SNPs 1_69925817 and 1_70066309 in MQTL2 interval, 4_211335022 in MQTL14 interval, 6_105377877 in MQTL24 interval, and 8_113534052 in MQTL28 interval. Three significance SNPs were not within the MQTL interval, but were very close to MQTL. These included SNPs 5_193598872, which was close to MQTL20 (451 kb); 5_212791755, which was close to MQTL21 (306 kb); and 9_93664580, which was close to MQTL32 (687 kb) (Figure 5). Additionally, 5_212791755 was a co-location SNP in two environments. These results further verified the accuracy of the SNPs related to ear traits in this study.

## 3. Discussion

### 3.1. Genetic Basis of Ear Traits Related to Yield

Grain yield is one of the most important aspects of agricultural production, and high yield is one of the most important breeding goals that breeders strive to pursue throughout their lives. Maize yield is affected by a variety of agronomic traits and is one of the most complex traits determined by many factors. Maize ear traits are composed of ear inflorescence structure and are highly correlated with yield [19]. In-depth study of the genetic basis and molecular mechanism of ear traits is beneficial to the improvement of yield. At present, some ear trait-related genes involved in yield have been studied. For example, the *krn1* located by Wang et al. through map-based cloning changes maize ERN by affecting the spikelet meristem [20]; Liu et al. located *KRN4* to the intergenic region 3 kb downstream of the SBP-box-containing gene UB to regulate the ERN [21]; *ZmGW2* encodes a RING-domain E3 ubiquitin ligase that regulates HKW in maize [22]; *ZmBAM1d* encodes a CLAVATA1 (CLV1)/BARELY ANY MERISTEM (BAM)-related receptor kinase-like protein, and overexpression of this gene increases HKW in maize [23]. Ear traits are complex traits affected by multiple factors, and there are few studies on the related genetic basis analysis and excellent functional genes. Therefore, more work needs to be performed to study the genetic basis of maize ear development.

Owing to the complexity of grain yield genetics, it has often replaced by ear traits in genetic studies [9]. In this study, correlation analysis of ear traits showed that yield was mainly related to ERN, KNR, ED, EL and HKW, which was consistent with the research results of Upadyayula et al. and Sivakumar et al. [19,24]. The heritability of ear traits was between 70.25% and 89.04%, indicating that ear-related traits were mainly controlled by genetic effects, similar to the results found in most other studies [25,26]. Different studies show, different levels of heritability of each trait, which may be due to various reasons, such as different test materials, environmental differences, and different data survey methods. In this study, the heritability of each ear trait was relatively high, which was helpful for analyzing the genetic basis of ear traits in different environments.

### 3.2. Comparison of Different Association Analysis Models

GWAS is based on linkage disequilibrium, combined with high-quality SNPs in the whole genome of a species, using different statistical models to analyze the genetic basis of target traits. The accuracy of GWAS results is affected by the statistical efficacy, which is also affected by various factors, such as the size of the associated population, the type of population, the marker density and the statistical model. Therefore, when the population size, the type of population and the marker density are certain, the accuracy of GWAS analysis results can be improved by selecting the optimal model. Yang et al. found that the GWAS of maize was greatly affected by population structure and kinship; therefore, it is necessary to select the best statistical model to study the relationship between genotypes and different traits, so as to improve the statistical efficacy of GWAS and ensure the accuracy of the results [27]. In order to improve the statistical efficacy of GWAS and minimize the impact of false positives and false negatives on the results of association analysis, this study used GLM, MLM, MLMM, BLINK and FarmCPU models for GWAS. The FarmCPU model had the better ability to control false positive and false negative, and had higher statistical efficacy, making it the best model in this study. This result is similar to those of some other studies, such as Liu et al., who found that the FarmCPU model had the highest detection efficiency [28]. Kaler et al. compared eight statistical models for association mapping of three empirical phenotypic traits. The research showed that the FarmCPU was an appropriate model for controlling false positives and false negatives compared to other models [29]. Because the results of GWAS will be affected by various factors, an excellent statistical model of association analysis is very important to reduce the interference of various effects, to detect micro effect loci and genes, and to ensure the accuracy of association analysis results, which is of great significance to the research results of GWAS.

### 3.3. Comparison of Co-Localization SNPs with Previous Localization Results

We found ten co-localized markers. Of these, SNPs 3_194720972 was located in bin3.07, and this position is similar to SNPs for ERN found by Liu et al. [30]. SNPs 5_212791755 was located in bin5.08, which is similar to SNPs for ERN found by Zhang et al. [3]. SNPs 3_218404753 was located in bin3.09, which is the same bin in which the ED-related SNPs (PZE_103171163) were found by Zhu et al. through GWAS [25]. The detection of SNPs locations that are similar to those found in previous research can explain to some extent the accuracy of the results in this study. However, some newly detected SNPs were found to be inconsistent with the previous research results, which may be due to different materials, methods, experimental environments, etc., resulting in inconsistent positioning results. For five of the eight ear traits in this study, including ERN, ED, EW, HKW and VW, we repeatedly detected co-localized SNPs in more than two environments or between two traits, but no co-localization SNPs were detected for KNR, CD and EL traits. It was speculated that KNR, CD and EL traits were more affected by the external environment than the other five traits. Many significant SNPs were detected on ear traits in different years, but less co-located SNPs were detected in multiple environments. Ear traits are mainly quantitative traits controlled by multiple genes, which are greatly affected by the environment. Therefore, different environmental conditions may cause differential expression the genes controlling these traits. The genes detected in multiple environments were stably expressed, and there were relatively few co-location SNPs in multiple environments. On the other hand, this may be related to the relatively small number and low density of high-quality SNPs (31,826) after quality control.

### 3.4. Functional Prediction Analysis of Candidate Genes

This study predicted nine candidate genes most likely to be associated with the trait. *Zm00001d027326* encodes an actin-related protein like 4 (ARP4). ARP4 is involved in various biological processes by interacting with *GIF1*, and *GIF1* controls ear inflorescence architecture and floral development by regulating key genes in hormone biosynthesis and meristem determinacy in maize [31]. In *Arabidopsis*, *POD1* regulates the pollen tube response to signals from the female tissues during pollen tube guidance and early embryo patterning [32]. *Zm00001d013172* encodes bZIP-transcription factor55 (bZIP55). The basic leucine zipper-like transcription factor is one of the most studied transcription factor gene families in plants [33]. In maize, Seetharam et al. associated *Zm00001d013172* with yield under heat stress conditions [34]. *Zm00001d043292* encodes the Transducin/WD40 repeat-like superfamily protein. WD repeat proteins are proteins containing multiple conserved WD motifs (WD40), which play important roles in signal transduction, protein transport, and RNA processing [34]. In *Arabidopsis*, WD40 repeat protein gene mutant *at1g65030* showed an increase in seed weight and volume, but the seed setting rate of silique seeds was reduced. The overexpression of this gene resulted in reduced seed weight. In maize, Chen et al. identified the *KRN2* gene encoding the WD40 protein to control the number of maize kernels, and knockout of *KRN2* increased grain yield by about 10% [35]. *Zm00001d044069* (*spi1*) encodes a flavonoid monooxygenase, similar to the *YUCCA* (*YUC*) genes of *Arabidopsis*, which are involved in local auxin biosynthesis in various plant tissues, catalyzing the rate-limiting step in tryptophan-dependent auxin biosynthesis [36]. Throughout the nutritional and reproductive development of maize, *spi1* plays a role in the formation of axillary meristems and lateral organs; *spi1* mutants’ tassels have fewer branches and spikelets, and ears are small with fewer kernels [37]. *Zm00001d022049* encodes pentapeptide repeat protein 408 (PPR408). PPR proteins play an important role in regulating embryogenesis, and plant growth and development. Many PPR gene mutations have lethal effects, and mutants show abnormal embryonic development [38]. Mutations in the maize PPR gene *EMP5* result in reduced mature seed volume and loss of the embryo or endosperm [39]. Mutations in *MPPR6* cause a quarter of the inner core to be smaller, delay embryonic development, and reduce amyloid layers [40]. *Zm00001d036455* encodes the leucine-rich repeat (LRR) family protein, which plays an important role in plant growth, development, and disease resistance. Leucine-rich repeats receptor-like protein kinase (LRR-RLK) belong to the LRR protein family. LRR-RLK has important regulatory functions in plant growth and development, hormone signal transduction, and biotic and abiotic stress responses [41]. The *Arabidopsis CLV1* gene encodes LRR-RLK, and the mutation in *CLV1* results in plants with enlarged apical and flower meristems, extra floral organs and a loss of flower meristem determinacy [42]. In maize, mutations in the LRR-like receptor protein kinase gene *td1*, which is homologous to *Arabidopsis CLV1*, lead to the expansion of floral meristems and abnormal heading, which eventually leads to the clustering of ear tips [43]. *Zm00001d047736* encodes an asparagine synthase 4 (ASN4). Nitrogen is one of the most important elements in plant nutrition and plays a key role in improving crop growth and yield [44]. Therefore, ASN plays a crucial role in nitrogen metabolism and transport in plants [45]. In *Arabidopsis*, overexpression of *AtASN1* and *AtASN2* increased seed protein content and glutamine content, respectively [46]. In maize, *ZmASN4* is involved in the gene regulatory network of maize nitrogen use efficiency and maize response to nitrogen [47,48]. *Zm00001d042314* encodes a CBS domain-containing protein CBSCBSPB1. The CBS domain has no defined functions, but plays a regulatory role for many enzymes [49]. In rice, the *DPS1* gene encodes a CBS domain protein, and *DPS1* plays a key role in panicle and anther development and controls rice seed setting [50]. The CBS domain protein mainly plays a role in abiotic and biotic stress responses [51]. *OsCBSCBSPB4* is involved in abiotic stress responses, improving a variety of abiotic stress tolerances, delaying leaf senescence, promoting root growth and increasing biomass [52]. Among the nine candidate genes, *Zm00001d044069* was verified in maize to be related to the reduction in ear grain, and the other eight candidate genes related to ear traits were also found to be likely related to the development of maize yield and ear traits through functional prediction analysis, which has certain significance for gene function verification research.

### 3.5. Analyzing the Genetic Basis of Ear Traits by Combining GWAS and Meta-QTL

Despite many QTL related to yield identified in maize, very few of them have been useful in genetic improvement programs due to their minor effects and the influence of the environment. It is a normal phenomenon that QTL discovered using one mapping population do not truly work well in a breeding program that involves a different population. Reanalysis of already identified QTL through meta-analysis of QTL is one of the most promising methodologies for the integration of QTL and the prediction of stable and robust MQTL. Meta-analysis combines data from several QTL mapping studies in diverse environments and different genetic backgrounds to identify stable, major and reliable MQTL with reduced CI [53]. Therefore, the main objective of MQTL analysis is to identify stable QTL in the genomes, which can be useful in breeding programs through marker-assisted selection [18]. The ultimate goal of all the breeding programs is to achieve a high-grain yield in different environments [11].

Out of the 41 MQTL identified in this study, 19 MQTL have detected functional genes and candidate genes for yield-related ear traits that have been reported in maize, indicating that meta-analysis is an efficient and feasible method for mining genes for yield-related traits in maize. The five significant SNPs identified by GWAS in this study fell within the MQTL interval, and the three significant SNPs were close to the MQTL (less than 1 Mb). The significant SNPs (identified in GWAS) within/around the MQTL region confirms the presence of yield candidate genes in such genomic regions. These SNPs and intervals validated by GWAS and MQTL should be prioritized for identifying candidate genes controlling ear traits in maize, and validated through expression studies of relevant germplasm. The mutual validation between GWAS and MQTL demonstrates the accuracy of the results of this study. The lack of verification for the remaining MQTL with significant SNPs can be attributed to the diversity of the genetic material across studies. It may also indicate that the QTL or SNPs identified in mapping studies could be cultivar-specific and not shared across another germplasm [14]. Therefore, the results of this study, combined with GWAS and meta-analysis, will provide important information for further precise mapping of yield-related genes, thus revealing the corresponding molecular mechanisms.

GWAS and Meta-QTL only analyze the genetic basis of maize ear traits from genomics, which has certain limitations. Yield-related traits are complex traits that are highly correlated with population structure. Identifying regulatory genes related to target traits using GWAS and QTL is easily influenced by population structure [54], and the detection ability of quantitative traits controlled by some micro effect multiple genes is insufficient. It is difficult to accurately identify micro effect sites or genes, and false positive or false negative results may be introduced during the detection process, which may lead to inaccurate experimental results. Next, joint analysis should be conducted on genomics, transcriptomics, proteomics, metabolomics, etc. The analysis of multiple omics is an effective way to obtain key genes for different traits and an effective strategy to analyze the genetic basis of different traits, because it can mutually compensate for the shortcomings of each method [55,56]. Multi-omics methods integrate information from several omics levels, providing more evidence for biological mechanisms.

## 4. Materials and Methods

### 4.1. Material and Experimental Design

A total of 580 maize inbred lines were analyzed in the study. These were derived from the Canadian early maturing improved group, BSSS, NSS, P group, Huanghuaihai group, European KWS series and Pioneer series, and provided by the Crop Institute of the Xinjiang Academy of Agricultural and Reclamation Sciences. In 2019, 2020 and 2021, they were planted in the maize breeding test field of the Crop Research Institute, Xinjiang Academy of Agricultural Sciences, Shihezi (44.31° N, 85.99° E), with a planting density of 105,000 plants/ha. The field design adopted α Latin square design with 2 repetitions, each maize inbred line was grown in a single row (row length 4.5 m, row spacing 0.55 m), and field management was the same as that of local field production management.

### 4.2. Phenotypic Trait Determination

After harvesting, the ear phenotypic traits were measured. Four uniform ears were randomly selected and the number of ear rows in the middle of the ear and the number of kernels in one row were counted to determine ERN and KNR. Eight uniform ears were selected to measure EL, ED, CD and EW, eight ears were arranged head to tail in a column to measure the length from the bottom to the top of the ears and the average value was taken as EL. Eight ears lined vertically with head and tail phased, then the middle diameter of the ears was measured and the average value was taken as ED. Eight cobs were closely arranged to measure the overall diameter of the middle of the cob and the average value was taken as CD. Eight uniform ears were selected to measure the total weight and the average value was taken as EW. One hundred seeds were randomly selected and weighed as HKW, and the measurement was repeated 2 times. After ear threshing, the grain moisture meter was used to measure the VW and moisture 2 times. EW, HKW and VW were converted according to standard moisture (14%).

### 4.3. Phenotypic Data Analysis

Descriptive statistical analysis, variance analysis and correlation analysis were performed on the phenotypic data using R 4.1.3 software, and the corresponding graphs were drawn. Heritability was calculated according to the proposed formula $H^{2}=\sigma_{g}^{2}/\left(\sigma_{g}^{2}+\sigma_{gy}^{2}/n+\sigma_{e}^{2}/\mathrm{nr}\right)$ [57], where $\sigma_{g}^{2}$ is the genetic variance, $\sigma_{gy}^{2}$ is the variance of the interaction between genotype and year, $\sigma_{e}^{2}$ is the error variance, n is the number of years, and r is the number of repetitions. To eliminate the influence of environmental (year) variation in phenotypic values, a mixed linear model was constructed using the R package lme4 to estimate the BLUE of eight ear traits for subsequent GWAS.

### 4.4. Genotyping and Population Structuredetermination

Genomic DNA was extracted from the fresh leaves by a modified CTAB method, and the quality of DNA extraction was tested. SNPs genotyping data were obtained using Maize SNPs 40 K GBTS technology. The SNPs with minor allele frequency (MAF) > 0.05 and missing rate < 0.05 were retained by PLINK1.9. Finally, 31,826 high-quality SNPs were retained to conduct GWAS. The population structure was estimated using the STRUCTURE 2.3.4 software program [58], the number of subgroups (K) was set from 1 to 10, and each K value was repeated 10 times. The length of the burn-in period was set to 10,000, and Markov Chain Monte Carlo (MCMC) was set to 10,000 and conducted using the mixture model and correlated allele frequency for each K. Based on the output log likelihood of data (LnP(D)) of STRUCTURE 2.3.4, the ad hoc statistic ∆K was applied to determine the reasonable subgroups number; the results of multiple runs were integrated using the CLUMPP 1.1.2 software and plotted using R 4.1.3 software. PCA, kinship and LD decay were calculated using Tassel 5.0.

### 4.5. Genome-Wide Association Study

The association analysis of the BLUE of ear traits was performed by the GLM, MLM, MLMM, BLINK and FarmCPU models of the GAPIT package of R 4.1.3 software, and the false positives and false negatives of these five models were evaluated based on the QQ plots to find the best model for subsequent GWAS. PCA and kinship were used as covariates in the association analysis to reduce false positives. To control for the probability of false positives, the threshold was adjusted using the Bonferroni correction (*p* = 0.05/N, N is the total number of SNPs). The value *p* = 1.57 × 10^−6^ was used as the threshold level line for significant correlation to select significant SNPs (the VW only has phenotypic data in 2020 and 2021; according to the overall GWAS analysis of VW, Bonferroni correction was too strict for VW, so *p* = 1 × 10^−4^ was taken as the threshold for the VW).

### 4.6. Candidate Gene Mining and Functional Analysis

Based on the physical positions of the SNPs, which were significantly associated with target traits, and chromosome average LD decay distance (*r*^2^ = 0.1), the MaizeGDB (https://maizegdb.org/gbrowse/maize_v4) (accessed on 20 September 2022) database (B73_RefGen_v4) was used for identifying candidate genes and the functional annotations. RNA_seq data of B73 inbred line ear development-related tissue sites were downloaded from MaizeGDB for candidate gene tissue-specific expression analysis. Candidate genes were functionally annotated and predicted using MaizeGDB and NCBI (https://www.ncbi.nlm.nih.gov/) (accessed on 20 September 2022).

### 4.7. Collection of QTL Information Related to Ear Traits of Maize

Using keywords such as maize, yield, ear, QTL, and high-density genetic map from the Web of Science website (http://www.webofknowledge.com/) (accessed on 1 July 2023) and CNKI (https://www.cnki.net/) (accessed on 1 July 2023), more than 30 related articles from 2005 to 2023 were retrieved. The articles providing QTL intervals and flanking markers were selected for QTL collection (Table S8). For the experiments with a complete genetic map and QTL information, the information was arranged according to the format required by the software; the information on all essential parameters like QTL name, position, traits, linkage group, LOD (Likelihood odd) values, CI, phenotypic variance explained (R^2^), etc., were used for the preparation of QTL files.

### 4.8. QTL Projection and Meta-Analysis

Map projection and the following meta-analysis were implemented by the BioMercator 4.2.3 software [59]. Collected maize yield QTL were iteratively projected onto the target map IBM2 2008 Neighbors, and MQTL analysis was carried out on the QTL clusters present on each chromosome. In this method, all the possible QTL combinations were tested based on the QTL model, AIC (Akaike information content), AICc (AIC correction), AIC3 (AIC 3 candidate models), BIC (Bayesian information criterion), and AWE (Average weight of evidence), and the model with the lowest AIC represents the number of MQTL, and the initial number of QTL used for meta-QTL was not less than three [25]. Further, the position and CI (95%) of the MQTL were calculated and the flanking markers for MQTL were identified from MaizeGDB. After obtaining MQTL given by the software, the physical location of partial markers on B73 (AGPv4) of IBM2 2008 Neighbors could be found on MaizeGDB. Finally, the MaizeGDB was used to look up the genes contained in MQTL as candidate genes.

## 5. Conclusions

In this study, the FarmCPU model was the best model. A total of 104 significantly associated SNPs were detected for eight ear traits, and 10 co-localized SNPs were repeatedly detected in at least two environments or between two traits. Nine candidate genes were predicted to be most likely related to the ear traits. Meta-analysis of yield-related ear traits identified 41 MQTL, and 29 yield-related ear trait functional genes and candidate genes reported in maize were detected in 19 MQTL. Five significant SNPs detected by GWAS were located within these MQTL intervals, and another three significant SNPs were close to MQTL. The combined analysis of GWAS and Meta-QTL ensured that the experimental results were accurate and reliable, and provided reference for resolving the genetic basis of maize yield-related traits in terms of both SNPs and QTL. The results of this study will provide important information for maize yield-related gene cloning and breeding programs.

## Figures and Tables

**Table 1:** Analysis of variance of ear traits.

Source	ERN	KNR	EL	ED	CD	EW	HKW	VW
Genotype	17.13 ***	96.4 ***	18.5 ***	0.86 ***	0.31 ***	3301 ***	116.5 ***	2174 ***
Year	45.04 ***	377.9 ***	537 ***	23.21 ***	1.74 ***	189,927 ***	1799.2 ***	32,831 ***
Genotype × Year	2.1 ***	22.1 ***	3.5 ***	0.12 ***	0.04 ***	689 ***	13 ***	717
Residual	1.26	11.6	1.8	0.06	0.02	302	6.6	656
*H*^2^ (%)	87.71	76.74	86.45	85.69	80.41	80.82	89.04	70.25

ERN, ear row number; KNR, kernel number per row; EL, ear length; ED, ear diameter; CD, cob diameter; EW, ear weight; HKW, hundred kernel weight; VW, volume weight. *H*^2^, heritability. The analysis of variance results in the table are represented as mean square (MS). *** Significant levels at *p* < 0.001.

**Table 2:** Significant colocalization of SNPs for ear traits.

Trait	SNPs	Chromosome	Position (bp)	Allele	*p* Value			
					2019	2020	2021	BLUE
ERN	1_3069018	1	3,069,018	T/G		9.69 × 10^−7^		1.10 × 10^−6^
ERN	5_212791755	5	212,791,755	C/T	1.88 × 10^−10^	2.04 × 10^−7^		
ED	5_5898430	5	5,898,430	C/T			7.54 × 10^−8^	5.07 × 10^−7^
ERN, ED	3_194720972	3	194,720,972	T/C		5.11 × 10^−7^		
						1.88 × 10^−9^		
ERN, ED	3_218404753	3	218,404,753	T/C		1.23 × 10^−8^	9.64 × 10^−11^	
ERN, ED	7_168679099	7	168,679,099	T/C				6.79 × 10^−9^
								9.14 × 10^−7^
EW	6_88904986	6	88,904,986	T/A	7.15 × 10^−8^			5.03 × 10^−7^
EW	9_140213959	9	140,213,959	T/C			1.68 × 10^−7^	6.98 × 10^−7^
EW, HKW	9_75603144	9	75,603,144	C/T			1.31 × 10^−7^	8.10 × 10^−7^
VW	3_160707766	3	160,707,766	G/A			9.18 × 10^−5^	9.44 × 10^−5^

ERN, ear row number; ED, ear diameter; EW, ear weight; HKW, hundred kernel weight; VW, volume weight; BLUE, best linear unbiased estimate.

**Table 3:** Candidate gene functional annotation.

Trait	SNPs	Gene	Annotation
ERN	1_3069018	*Zm00001d027326*	actin related protein like4
ERN	5_212791755	*Zm00001d018046*	Protein POLLEN DEFECTIVE IN GUIDANCE 1
ED	5_5898430	*Zm00001d013172*	bZIP-transcription factor55
ERN, ED	3_194720972	*Zm00001d043292*	Transducin/WD40 repeat-like superfamily protein
ERN, ED	3_218404753	*Zm00001d044069*	sparse inflorescence1
ERN, ED	7_168679099	*Zm00001d022049*	pentatricopeptide repeat protein408
EW	6_88904986	*Zm00001d036455*	Leucine-rich repeat (LRR) family protein
EW	9_140213959	*Zm00001d047736*	asparagine synthetase4
VW	3_160707766	*Zm00001d042314*	CBS domain-containing protein CBSCBSPB1

ERN, ear row number; ED, ear diameter; EW, ear weight; VW, volume weight.

## Data Availability

Data are contained within the article and Supplementary Materials.

## Supplementary Materials

The following supporting information can be downloaded at: https://www.mdpi.com/article/10.3390/plants12223806/s1, Figure S1: QQ plots of GWAS in the five models; Figure S2: Manhattan map and QQ map of ear traits in different years; Figure S3: Significance analysis of allele difference of Co-located SNP in different years; Figure S4: Analysis of allelic effects of colocalized SNPs for ear-related traits; Figure S5: Effect of superior allele number on traits; Figure S6: Heatmap of tissue-specific expression patterns of candidate genes; Figure S7: Initial QTL distribution on 10 chromosomes of ear traits; Figure S8: Projection and distribution of QTL and MQTL (Meta QTL) identified for ear traits. Table S1: Descriptive statistics of ear traits; Table S2: Correlation analysis of ear traits; Table S3: Significant SNP for ear traits; Table S4: Co-location of SNP interval candidate genes; Table S5: Gene expression of candidate genes related to traits in different tissues in public database; Table S6: Description of MQTL identified in the present study for ear traits in maize; Table S7: Candidate genes detected by meta-analysis; Table S8: Ear traits QTL mapped from 39 papers. References [1,25,60,61,62,63,64,65,66,67,68,69,70,71,72,73,74,75,76,77,78,79,80,81,82,83,84,85,86,87,88,89,90,91,92,93,94,95,96,97,98,99,100,101,102,103,104,105,106,107,108,109,110,111,112,113,114,115,116,117,118,119,120,121,122] are cited in the Supplementary Materials.

## References

[1] Liu J., Huang J., Guo H., Lan L., Wang H., Xu Y., Yang X., Li W., Tong H., Xiao Y. (2017). The Conserved and Unique Genetic Architecture of Kernel Size and Weight in Maize and Rice. Plant Physiol..

[2] Ren H., Liu M., Zhang J., Liu P., Liu C. (2022). Effects of agronomic traits and climatic factors on yield and yield stability of summer maize (*Zea mays* L.) in the Huang-Huai-Hai Plain in China. Front. Plant Sci..

[3] Zhang C., Zhou Z., Yong H., Zhang X., Hao Z., Zhang F., Li M., Zhang D., Li X., Wang Z. (2017). Analysis of the genetic architecture of maize ear and grain morphological traits by combined linkage and association mapping. Theor. Appl. Genet..

[4] Aranzana M.J., Kim S., Zhao K., Bakker E., Horton M., Jakob K., Lister C., Molitor J., Shindo C., Tang C. (2005). Genome-wide association mapping in Arabidopsis identifies previously known flowering time and pathogen resistance genes. PLoS Genet..

[5] Xiao Y., Liu H., Wu L., Warburton M., Yan J. (2017). Genome-wide Association Studies in Maize: Praise and Stargaze. Mol. Plant.

[6] An Y., Chen L., Li Y.X., Li C., Shi Y., Zhang D., Li Y., Wang T. (2020). Genome-wide association studies and whole-genome prediction reveal the genetic architecture of KRN in maize. BMC Plant Biol..

[7] Zhang X., Ren Z., Luo B., Zhong H., Ma P., Zhang H., Hu H., Wang Y., Zhang H., Liu D. (2022). Genetic architecture of maize yield traits dissected by QTL mapping and GWAS in maize. Crop J..

[8] Brown P.J., Upadyayula N., Mahone G.S., Tian F., Bradbury P.J., Myles S., Holland J.B., Flint-Garcia S., McMullen M.D., Buckler E.S. (2011). Distinct genetic architectures for male and female inflorescence traits of maize. PLoS Genet..

[9] Yang L., Li T., Tian X., Yang B., Lao Y., Wang Y., Zhang X., Xue J., Xu S. (2020). Genome-wide association study (GWAS) reveals genetic basis of ear-related traits in maize. Euphytica.

[10] Zhang X., Guan Z., Wang L., Fu J., Zhang Y., Li Z., Ma L., Liu P., Zhang Y., Liu M. (2020). Combined GWAS and QTL analysis for dissecting the genetic architecture of kernel test weight in maize. Mol. Genet. Genom..

[11] Wang Y., Huang Z., Deng D., Ding H., Zhang R., Wang S., Bian Y., Yin Z., Xu X. (2012). Meta-analysis combined with syntenic metaQTL mining dissects candidate loci for maize yield. Mol. Breed..

[12] Goffinet B., Gerber S. (2000). Quantitative Trait Loci: A Meta-analysis. Genetics.

[13] Gopinath I., Muthusamy V., Katral A., Zunjare R.U., Madhavan J., Yathish K.R., Sekhar J.C., Hossain F. (2023). Meta-QTL analysis and identification of candidate genes governing popping quality attributes in maize. South Afr. J. Bot..

[14] Gupta M., Choudhary M., Singh A., Sheoran S., Singla D., Rakshit S. (2023). Meta-QTL analysis for mining of candidate genes and constitutive gene network development for fungal disease resistance in maize (*Zea mays* L.). Crop J..

[15] Guo Z.F., Wang H.W., Tao J.J., Ren Y.H., Xu C., Wu K.S., Zou C., Zhang J.A., Xu Y.B. (2019). Development of multiple SNP marker panels affordable to breeders through genotyping by target sequencing (GBTS) in maize. Mol. Breed..

[16] Stelpflug S.C., Sekhon R.S., Vaillancourt B., Hirsch C.N., Buell C.R., de Leon N., Kaeppler S.M. (2016). An Expanded Maize Gene Expression Atlas based on RNA Sequencing and its Use to Explore Root Development. Plant Genome.

[17] Walley J.W., Sartor R.C., Shen Z., Schmitz R.J., Wu K.J., Urich M.A., Nery J.R., Smith L.G., Schnable J.C., Ecker J.R. (2016). Integration of omic networks in a developmental atlas of maize. Science.

[18] Arriagada O., Arévalo B., Cabeza R.A., Carrasco B., Schwember A.R. (2022). Meta-QTL Analysis for Yield Components in Common Bean (*Phaseolus vulgaris* L.). Plants.

[19] Upadyayula N., Da Silva H.S., Bohn M.O., Rocheford T.R. (2006). Genetic and QTL analysis of maize tassel and ear inflorescence architecture. Theor. Appl. Genet..

[20] Wang J., Lin Z., Zhang X., Liu H., Zhou L., Zhong S., Li Y., Zhu C., Lin Z. (2019). krn1, a major quantitative trait locus for kernel row number in maize. New Phytol..

[21] Liu L., Du Y., Shen X., Li M., Sun W., Huang J., Liu Z., Tao Y., Zheng Y., Yan J. (2015). KRN4 Controls Quantitative Variation in Maize Kernel Row Number. PLoS Genet..

[22] Li Q., Li L., Yang X., Warburton M.L., Bai G., Dai J., Li J., Yan J. (2010). Relationship, evolutionary fate and function of two maize co-orthologs of rice GW2 associated with kernel size and weight. BMC Plant Biol..

[23] Yang N., Liu J., Gao Q., Gui S., Chen L., Yang L., Huang J., Deng T., Luo J., He L. (2019). Genome assembly of a tropical maize inbred line provides insights into structural variation and crop improvement. Nat. Genet..

[24] Sivakumar S., Dhasarathan M., Karthikeyan A., Bharathi P., Kumari Vinodhana N., Ganesamurthy K., Senthil N. (2019). Population structure and association mapping studies for yield-related traits in Maize (*Zea mays* L.). Curr. Plant Biol..

[25] Zhu X.M., Shao X.Y., Pei Y.H., Guo X.M., Li J., Song X.Y., Zhao M.A. (2018). Genetic Diversity and Genome-Wide Association Study of Major Ear Quantitative Traits Using High-Density SNPs in Maize. Front. Plant Sci..

[26] Xiao Y., Tong H., Yang X., Xu S., Pan Q., Qiao F., Raihan M.S., Luo Y., Liu H., Zhang X. (2016). Genome-wide dissection of the maize ear genetic architecture using multiple populations. New Phytol..

[27] Yang X., Yan J., Shah T., Warburton M.L., Li Q., Li L., Gao Y., Chai Y., Fu Z., Zhou Y. (2010). Genetic analysis and characterization of a new maize association mapping panel for quantitative trait loci dissection. Theor. Appl. Genet..

[28] Liu M., Tan X.L., Yang Y., Liu P., Zhang X.X., Zhang Y.C., Wang L., Hu Y., Ma L.L., Li Z.L. (2020). Analysis of the genetic architecture of maize kernel size traits by combined linkage and association mapping. Plant Biotechnol. J..

[29] Kaler A.S., Gillman J.D., Beissinger T., Purcell L.C. (2019). Comparing Different Statistical Models and Multiple Testing Corrections for Association Mapping in Soybean and Maize. Front. Plant Sci..

[30] Liu L., Du Y., Huo D., Wang M., Shen X., Yue B., Qiu F., Zheng Y., Yan J., Zhang Z. (2015). Genetic architecture of maize kernel row number and whole genome prediction. Theor. Appl. Genet..

[31] Li M., Zheng Y., Cui D., Du Y., Zhang D., Sun W., Du H., Zhang Z. (2022). GIF1 controls ear inflorescence architecture and floral development by regulating key genes in hormone biosynthesis and meristem determinacy in maize. BMC Plant Biol..

[32] Li H.J., Xue Y., Jia D.J., Wang T., Hi D.Q., Liu J., Cui F., Xie Q., Ye D., Yang W.C. (2011). POD1 regulates pollen tube guidance in response to micropylar female signaling and acts in early embryo patterning in Arabidopsis. Plant Cell.

[33] Landschulz W.H., Johnson P.F., McKnight S.L. (1988). The leucine zipper: A hypothetical structure common to a new class of DNA binding proteins. Science.

[34] Seetharam K., Kuchanur P.H., Koirala K.B., Tripathi M.P., Patil A., Sudarsanam V., Das R.R., Chaurasia R., Pandey K., Vemuri H. (2021). Genomic regions associated with heat stress tolerance in tropical maize (*Zea mays* L.). Sci. Rep..

[35] Chen W.K., Chen L., Zhang X., Yang N., Guo J.H., Wang M., Ji S.H., Zhao X., Yin P., Cai L. (2022). Convergent selection of a WD40 protein that enhances grain yield in maize and rice. Science.

[36] Zhao Y., Christensen S.K., Fankhauser C., Cashman J.R., Cohen J.D., Weigel D., Chory J. (2001). A Role for Flavin Monooxygenase-Like Enzymes in Auxin Biosynthesis. Science.

[37] Gallavotti A., Barazesh S., Malcomber S., Hall D., Jackson D., Schmidt R.J., McSteen P. (2008). sparse inflorescence1 encodes a monocot-specific YUCCA-like gene required for vegetative and reproductive development in maize. Proc. Natl. Acad. Sci. USA.

[38] Sosso D., Mbelo S., Vernoud V., Gendrot G., Dedieu A., Chambrier P., Dauzat M., Heurtevin L., Guyon V., Takenaka M. (2012). PPR2263, a DYW-Subgroup Pentatricopeptide repeat protein, is required for mitochondrial nad5 and cob transcript editing, mitochondrion biogenesis, and maize growth. Plant Cell.

[39] Liu Y.J., Xiu Z.H., Meeley R., Tan B.C. (2013). Empty pericarp5 encodes a pentatricopeptide repeat protein that is required for mitochondrial RNA editing and seed development in maize. Plant Cell.

[40] Manavski N., Guyon V., Meurer J., Wienand U., Brettschneider R. (2012). An essential pentatricopeptide repeat protein facilitates 5′ maturation and translation initiation of rps3 mRNA in maize mitochondria. Plant Cell.

[41] Park S., Moon J.C., Park Y.C., Kim J.H., Kim D.S., Jang C.S. (2014). Molecular dissection of the response of a rice leucine-rich repeat receptor-like kinase (LRR-RLK) gene to abiotic stresses. J. Plant Physiol..

[42] Poulios S., Vlachonasios K.E. (2018). Synergistic action of GCN5 and CLAVATA1 in the regulation of gynoecium development in Arabidopsis thaliana. New Phytol..

[43] Bommert P., Lunde C., Nardmann J., Vollbrecht E., Running M., Jackson D., Hake S., Werr W. (2005). thick tassel dwarf1 encodes a putative maize ortholog of the Arabidopsis CLAVATA1 leucine-rich repeat receptor-like kinase. Development.

[44] Ye J.Y., Tian W.H., Jin C.W. (2022). Nitrogen in plants: From nutrition to the modulation of abiotic stress adaptation. Stress Biol..

[45] Gaufichon L., Reisdorf-Cren M., Rothstein S.J., Chardon F., Suzuki A. (2010). Biological functions of asparagine synthetase in plants. Plant Sci..

[46] Lam H.M., Wong P., Chan H.K., Yam K.M., Chen L., Chow C.M., Coruzzi G.M. (2003). Overexpression of the ASN1 gene enhances nitrogen status in seeds of Arabidopsis. Plant Physiol..

[47] Wani S.H., Vijayan R., Choudhary M., Kumar A., Zaid A., Singh V., Kumar P., Yasin J.K. (2021). Nitrogen use efficiency (NUE): Elucidated mechanisms, mapped genes and gene networks in maize (*Zea mays* L.). Physiol. Mol. Biol. Plants.

[48] Jiang L., Ball G., Hodgman C., Coules A., Zhao H., Lu C. (2018). Analysis of Gene Regulatory Networks of Maize in Response to Nitrogen. Genes.

[49] Kushwaha H.R., Singh A.K., Sopory S.K., Singla-Pareek S.L., Pareek A. (2009). Genome wide expression analysis of CBS domain containing proteins in *Arabidopsis thaliana* (L.) Heynh and Oryza sativa L. reveals their developmental and stress regulation. BMC Genom..

[50] Zafar S.A., Patil S.B., Uzair M., Fang J., Zhao J., Guo T., Yuan S., Uzair M., Luo Q., Shi J. (2020). Degenerated Panicle and Partial Sterility 1 (DPS1) encodes a cystathionine beta-synthase domain containing protein required for anther cuticle and panicle development in rice. New Phytol..

[51] Tomar S., Subba A., Bala M., Singh A.K., Pareek A., Singla-Pareek S.L. (2022). Genetic Conservation of CBS Domain Containing Protein Family in Oryza Species and Their Association with Abiotic Stress Responses. Int. J. Mol. Sci..

[52] Kumar R., Subba A., Kaur C., Ariyadasa T.U., Sharan A., Pareek A., Sopory S.K., Singla-Pareek S.L. (2018). OsCBSCBSPB4 is a Two Cystathionine-beta-Synthase Domain-containing Protein from Rice that Functions in Abiotic Stress Tolerance. Curr. Genom..

[53] Welcker C., Sadok W., Dignat G., Renault M., Salvi S., Charcosset A., Tardieu F. (2011). A Common Genetic Determinism for Sensitivities to Soil Water Deficit and Evaporative Demand: Meta-Analysis of Quantitative Trait Loci and Introgression Lines of Maize. Plant Physiol..

[54] Camus-Kulandaivelu L., Veyrieras J.B., Madur D., Combes V., Fourmann M., Barraud S., Dubreuil P., Gouesnard B., Manicacci D., Charcosset A. (2006). Maize adaptation to temperate climate: Relationship between population structure and polymorphism in the Dwarf8 gene. Genetics.

[55] Guo J., Li C., Zhang X., Li Y., Zhang D., Shi Y., Song Y., Li Y., Yang D., Wang T. (2020). Transcriptome and GWAS analyses reveal candidate gene for seminal root length of maize seedlings under drought stress. Plant Sci..

[56] Urrutia M., Blein-Nicolas M., Prigent S., Bernillon S., Deborde C., Balliau T., Maucourt M., Jacob D., Ballias P., Bénard C. (2021). Maize metabolome and proteome responses to controlled cold stress partly mimic early-sowing effects in the field and differ from those of Arabidopsis. Plant Cell Environ..

[57] Knapp S.J. (1986). Confidence intervals for heritability for two-factor mating design single environment linear models. Theor. Appl. Genet..

[58] Earl D.A., vonHoldt B.M. (2011). Structure Harvester: A website and program for visualizing STRUCTURE output and implementing the Evanno method. Conserv. Genet. Resour..

[59] Sosnowski O., Charcosset A., Joets J. (2012). BioMercator V3: An upgrade of genetic map compilation and quantitative trait loci meta-analysis algorithms. Bioinformatics.

[60] Sun H., Xu H., Li B., Shang Y., Wei M., Zhang S., Zhao C., Qin R., Cui F., Wu Y. (2021). The brassinosteroid biosynthesis gene, ZmD11, increases seed size and quality in rice and maize. Plant Physiol. Biochem..

[61] Baye W., Xie Q., Xie P. (2022). Genetic Architecture of Grain Yield-Related Traits in Sorghum and Maize. Int. J. Mol. Sci..

[62] Li Q., Yang X., Bai G., Warburton M.L., Mahuku G., Gore M., Dai J., Li J., Yan J. (2010). Cloning and characterization of a putative GS3 ortholog involved in maize kernel development. Theor. Appl. Genet..

[63] Brugière N., Zhang W., Xu Q., Scolaro E.J., Lu C., Kahsay R.Y., Kise R., Trecker L., Williams R.W., Hakimi S. (2017). Overexpression of RING Domain E3 Ligase ZmXerico1 Confers Drought Tolerance through Regulation of ABA Homeostasis. Plant Physiol..

[64] Wang C., Li H., Long Y., Dong Z., Wang J., Liu C., Wei X., Wan X. (2023). A Systemic Investigation of Genetic Architecture and Gene Resources Controlling Kernel Size-Related Traits in Maize. Int. J. Mol. Sci..

[65] Zhan J., Wang F., Xing W., Liu J., Fan Z., Tao Y. (2018). Fine mapping and candidate gene prediction of a major QTL for kernel number per ear in maize. Mol. Breed..

[66] Ren X., Pan Z., Zhao H., Zhao J., Cai M., Li J., Zhang Z., Qiu F. (2017). EMPTY PERICARP11 serves as a factor for splicing of mitochondrial nad1 intron and is required to ensure proper seed development in maize. J. Exp. Bot..

[67] Zaidi P.H., Shahid M., Seetharam K., Vinayan M.T. (2022). Genomic Regions Associated With Salinity Stress Tolerance in Tropical Maize (*Zea Mays* L.). Front. Plant Sci..

[68] Khatun M., Monir M.M., Lou X., Zhu J., Xu H. (2022). Genome-wide association studies revealed complex genetic architecture and breeding perspective of maize ear traits. BMC Plant Biol..

[69] Shen X., Zhao R., Liu L., Zhu C., Li M., Du H., Zhang Z. (2019). Identification of a candidate gene underlying qKRN5b for kernel row number in *Zea mays* L.. Theor. Appl. Genet..

[70] Zhou Z., Li G., Tan S., Li D., Weiß T.M., Wang X., Chen S., Würschum T., Liu W., Léon J. (2020). A QTL atlas for grain yield and its component traits in maize (*Zea mays*). Plant Breed..

[71] Luo Y., Zhang M., Liu Y., Liu J., Li W., Chen G., Peng Y., Jin M., Wei W., Jian L. (2022). Genetic variation in YIGE1 contributes to ear length and grain yield in maize. New Phytol..

[72] Liu L., Huang J., He L., Liu N., Du Y., Hou R., Du H., Qiu F., Zhang Z. (2019). Dissecting the genetic architecture of important traits that enhance wild germplasm resource usage in modern maize breeding. Mol. Breed..

[73] Choe E., Drnevich J., Williams M.M. (2016). Identification of Crowding Stress Tolerance Co-Expression Networks Involved in Sweet Corn Yield. PLoS ONE.

[74] Zhou G., Zhu Q., Yang G., Huang J., Cheng S., Yue B., Zhang Z. (2015). qEL7.2 is a pleiotropic QTL for kernel number per row, ear length and ear weight in maize (*Zea mays* L.). Euphytica.

[75] Ning Q., Jian Y., Du Y., Li Y., Shen X., Jia H., Zhao R., Zhan J., Yang F., Jackson D. (2021). An ethylene biosynthesis enzyme controls quantitative variation in maize ear length and kernel yield. Nat. Commun..

[76] Wang H.Q., Liu P., Zhang J.W., Zhao B., Ren B.Z. (2020). Endogenous Hormones Inhibit Differentiation of Young Ears in Maize (*Zea mays* L.) Under Heat Stress. Front. Plant Sci..

[77] Zhou Q., Fu Z., Liu H., Wang J., Guo Z., Zhang X., Tian R., Liu Y., Qu J., Li W. (2021). Mining novel kernel size-related genes by pQTL mapping and multi-omics integrative analysis in developing maize kernels. Plant Biotechnol. J..

[78] Zhang X., Guan Z., Li Z., Liu P., Ma L., Zhang Y., Pan L., He S., Zhang Y., Li P. (2020). A combination of linkage mapping and GWAS brings new elements on the genetic basis of yield-related traits in maize across multiple environments. Theor. Appl. Genet..

[79] Li S., Meng S., Weng J., Wu Q. (2022). Fine-tuning shoot meristem size to feed the world. Trends Plant Sci..

[80] Jia T.J., Li J.J., Wang L.F., Cao Y.Y., Ma J., Wang H., Zhang D.F., Li H.Y. (2020). Evaluation of drought tolerance in ZmVPP1-overexpressing transgenic inbred maize lines and their hybrids. J. Integr. Agric..

[81] Gong D., Wang Y., Zhang H., Liang K., Sun Q., Qiu F. (2023). Overexpression of ZmKL9 increases maize hybrid hundred kernel weight. Plant Biotechnol. J..

[82] Fei X., Wang Y., Zheng Y., Shen X., E L., Ding J., Lai J., Song W., Zhao H. (2022). Identification of two new QTLs of maize (*Zea mays* L.) underlying kernel row number using the HNAU-NAM1 population. BMC Genom..

[83] Ma Y., Li D., Xu Z., Gu R., Wang P., Fu J., Wang J., Du W., Zhang H. (2022). Dissection of the Genetic Basis of Yield Traits in Line per se and Testcross Populations and Identification of Candidate Genes for Hybrid Performance in Maize. Int. J. Mol. Sci..

[84] Liu X., Zheng Z., Tan Z., Li Z., He C. (2012). Quantitative trait locus (QTL) mapping for 100-kernel weight of maize (*Zea mays* L.) under different nitrogen regimes. Afr. J. Biotechnol..

[85] Zhang H., Zheng Z., Liu X., Li Z., He C., Liu D., Luo Y., Zhang G., Tan Z., Li R. (2010). QTL mapping for ear length and ear diameter under different nitrogen regimes in maize. Afr. J. Agric. Res..

[86] Chen J., Zhang L., Liu S., Li Z., Huang R., Li Y., Cheng H., Li X., Zhou B., Wu S. (2016). The Genetic Basis of Natural Variation in Kernel Size and Related Traits Using a Four-Way Cross Population in Maize. PLoS ONE.

[87] Zhao X., Peng Y., Zhang J., Fang P., Wu B. (2018). Identification of QTLs and Meta-QTLs for Seven Agronomic Traits in Multiple Maize Populations under Well-Watered and Water-Stressed Conditions. Crop Sci..

[88] Mei X., Dong E., Liang Q., Bai Y., Nan J., Yang Y., Cai Y. (2021). Identification of QTL for fasciated ear related traits in maize. Crop Sci..

[89] Liu X.H., Zheng Z.P., Tan Z.B., Li Z., He C. (2010). Genetic analysis of two new quantitative trait loci for ear weight in maize inbred line Huangzao4. Genet. Mol. Res..

[90] Yang C., Liu J., Rong T.Z. (2015). Detection of quantitative trait loci for ear row number in F2 populations of maize. Genetics and Mol. Res..

[91] Guo J.J., Chen Z.L., Liu Z.P., Wang B.B., Song W.B., Li W., Chen J., Dai J.R., Lai J.S. (2011). Identification of genetic factors affecting plant density response through QTL mapping of yield component traits in maize (*Zea mays* L.). Euphytica.

[92] Li Y.L., Li X.H., Li J.Z., Fu J.F., Wang Y.Z., Wei M.G. (2009). Dent corn genetic background influences QTL detection for grain yield and yield components in high-oil maize. Euphytica.

[93] Li M., Guo X., Zhang M., Wang X., Zhang G., Tian Y., Wang Z. (2010). Mapping QTLs for grain yield and yield components under high and low phosphorus treatments in maize (*Zea mays* L.). Plant Sci..

[94] Pan L., Yin Z., Huang Y., Chen J., Zhu L., Zhao Y., Guo J., Lübberstedt T. (2017). QTL for maize grain yield identified by QTL mapping in six environments and consensus loci for grain weight detected by meta-analysis. Plant Breed..

[95] Zhang Z., Shen T., Jiang T., Hu X., Wen M., Qiu H. (2022). Quantitative trait loci mapping of maize (*Zea mays*) ear traits under low-phosphorus stress. Plant Breed..

[96] Li Y.L., Niu S.Z., Dong Y.B., Cui D.Q., Wang Y.Z., Liu Y.Y., Wei M.G. (2007). Identification of trait-improving quantitative trait loci for grain yield components from a dent corn inbred line in an advanced backcross BC2F2 population and comparison with its F2:3 population in popcorn. Theor. Appl. Genet..

[97] Li J.Z., Zhang Z.W., Li Y.L., Wang Q.L., Zhou Y.G. (2011). QTL consistency and meta-analysis for grain yield components in three generations in maize. Theor. Appl. Genet..

[98] Liu Y., Wang L., Sun C., Zhang Z., Zheng Y., Qiu F. (2014). Genetic analysis and major QTL detection for maize kernel size and weight in multi-environments. Theor. Appl. Genet..

[99] Han X., Qin Y., Sandrine A.M.N., Qiu F. (2020). Fine mapping of qKRN8, a QTL for maize kernel row number, and prediction of the candidate gene. Theor. Appl. Genet..

[100] Yang C., Tang D., Zhang L., Liu J., Rong T. (2015). Identification of QTL for ear row number and two-ranked versus many-ranked ear in maize across four environments. Euphytica.

[101] Lan T.R., He K.H., Chang L.G., Cui T.T., Zhao Z.X., Xue J.Q., Liu J.C. (2018). QTL mapping and genetic analysis for maize kernel size and weight in multi-environments. Euphytica.

[102] Lu M., Xie C.X., Li X.H., Hao Z.F., Li M.S., Weng J.F., Zhang D.G., Bai L., Zhang S.H. (2011). Mapping of quantitative trait loci for kernel row number in maize across seven environments. Mol. Breed..

[103] Stange M., Schrag T.A., Utz H.F., Riedelsheimer C., Bauer E., Melchinger A.E. (2013). High-density linkage mapping of yield components and epistatic interactions in maize with doubled haploid lines from four crosses. Mol. Breed..

[104] Cai L., Li K., Yang X., Li J. (2014). Identification of large-effect QTL for kernel row number has potential for maize yield improvement. Mol. Breed..

[105] Shi Z., Song W., Xing J., Duan M., Wang F., Tian H., Xu L., Wang S., Su A., Li C. (2017). Molecular mapping of quantitative trait loci for three kernel-related traits in maize using a double haploid population. Mol. Breed..

[106] Yang C., Zhang L., Jia A., Rong T. (2016). Identification of QTL for maize grain yield and kernel-related traits. J. Genet..

[107] Jiang T., Zhang C., Zhang Z., Wen M., Qiu H. (2023). QTL mapping of maize (*Zea mays* L.) kernel traits under low-phosphorus stress. Physiol. Mol. Biol. Plants.

[108] Liu C., Zhou Q., Dong L., Wang H., Liu F., Weng J., Li X., Xie C. (2016). Genetic architecture of the maize kernel row number revealed by combining QTL mapping using a high-density genetic map and bulked segregant RNA sequencing. BMC Genom..

[109] Park K.J., Sa K.J., Kim B.W., Koh H.-J., Lee J.K. (2014). Genetic mapping and QTL analysis for yield and agronomic traits with an F2:3 population derived from a waxy corn × sweet corn cross. Genes Genom..

[110] Choi J.-K., Sa K.J., Park D.H., Lim S.E., Ryu S.-H., Park J.Y., Park K.J., Rhee H.-I., Lee M., Lee J.K. (2019). Construction of genetic linkage map and identification of QTLs related to agronomic traits in DH population of maize (*Zea mays* L.) using SSR markers. Genes Genom..

[111] Yang J., Liu Z., Chen Q., Qu Y., Tang J., Lübberstedt T., Li H. (2020). Mapping of QTL for Grain Yield Components Based on a DH Population in Maize. Scientific Reports..

[112] Lan J.H., Li X.H., Gao S.R., Zhang B.S., Zhang S.H. (2005). QTL Analysis of Yield Components in Maize under Different Environments. Acta Agron. Sin..

[113] Yan J.B., Tang H., Huang Y.Q., Zheng Y.L., Chander S., Li J.S. (2006). Genome wide scanning analysis of QTLs for major effects and epistasis in maize yield and component factors. Chin. Sci. Bull..

[114] Liu Z.H., Tang J.H., Wei X.Y., Wang C.L., Tian G.W., Hu Y.M., Chen W.C. (2007). QTL Mapping of Ear Traits under Low and High Nitrogen Conditions in Maize. Sci. Agric. Sin..

[115] Jin X.C., Li T.F., Wu F.Y., Wang X., Zhang L., Di H., Wang Z.H. (2011). QTL Mapping for Yield and Related Characters. J. Maize Sci..

[116] Zhang W.Q., Ku L.X., Zhang J., Han Z.P., Chen Y.H. (2013). QTL Analysis of Kernel Ratio; Kernel Depth; and 100-Kernel Weight in Maize (*Zea mays* L.). Acta Agron. Sin..

[117] Ren Z.Y., Su S.Z., Zhang S.Z., Liu H.L., Luo B.W., Liu D., Wu L., Rong T.Z., Gao S.B. (2015). Characterization and QTL Mapping of Yield Trait under Two Phosphorus Regimes in Maize. Acta Agric. Boreali-Sin..

[118] He K.H., Chang L.G., Li Y.N., Qu J.Z., Cui T.T., Xu S.T., Xue J.Q., Liu J.C. (2017). QTL mapping of ear traits of maize with and without N input. J. Plant Nutr. Fertil..

[119] Zhang Y.N., Zhang Q., Pan F.F., Cai X.Y., Zhang N., Li X., Huang X.Q. (2017). Detection of Quantitative Trait Loci for Maize Grain Yield and Kernel-related Traits. J. Fudan Univ. (Nat. Sci.).

[120] Zhao P.B., Jia X.Y., Ma J.R., Zhu L.Y., Zhao Y.F. (2022). Mapping and Analysis of QTLs Related to Ear Row Number in Maize Based on RIL Population. Seed.

[121] Chen H., Xue Y.D., Yang T.X., Yan P.S., Meng S.J., Wang L.L., Zhang Z.H. (2018). Identification QTL and heterotic loci for maize ear-related traits using a set of “immortalized F2” population of Nongda 108. J. Henan Agric. Univ..

[122] Zhao X.Q., Fang P., Peng Y.L., Gao Q.H., Zeng W.J., Ren B. (2018). QTL Mapping for Six Ear-related Traits Based on Two Maize (*Zea mays*) Related Populations. J. Agric. Biotechnol..

