# Supplementary material for: GWAS and Meta-QTL Analysis of Yield-Related Ear Traits in Maize
Source: Plants (Basel). 2023 Nov 8;12(22):3806. doi: 10.3390/plants12223806 (PMC10674677; doi:10.3390/plants12223806)
Supplement: Supplementary file 1 [file plants-12-03806-s001.zip › Supplementary Figure.pdf]

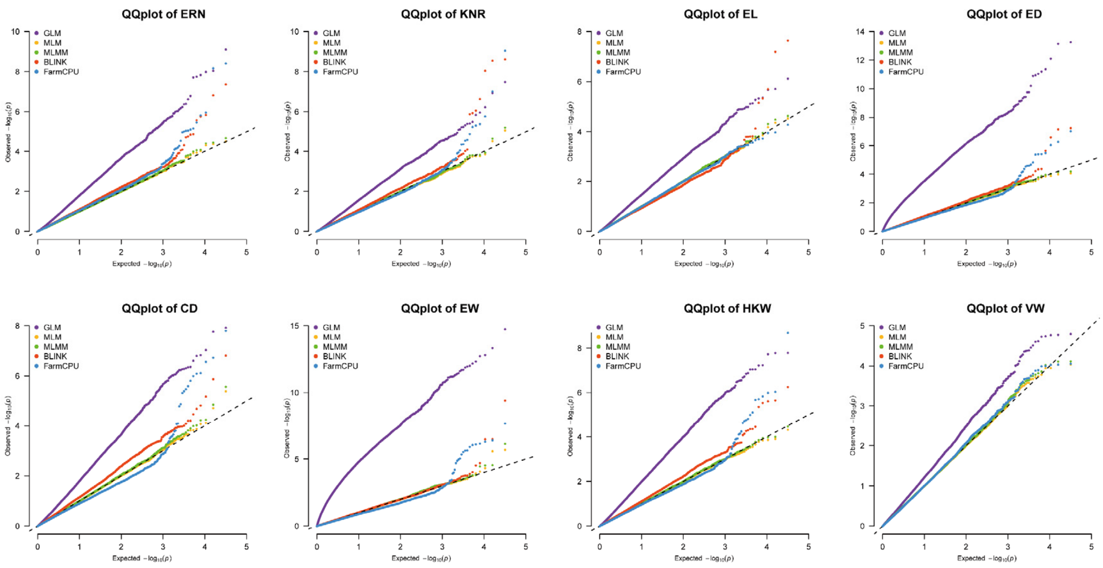

Figure S1. QQ plots of GWAS in the five models. ERN, ear row number; KNR, kernel number per row; EL, ear length; ED, ear diameter; CD, cob diameter; EW, ear weight; HKW, hundred kernel weight; VW, volume weight; GLM, General Linear Model; MLM, Mixed Linear Model; MLMM, Multiple Loci Mixed Model; BLINK, Bayesian-information and Linkage-disequilibrium Iteratively Nested Keyway; FarmCPU, Fixed and random model Circulating Probability Unification.

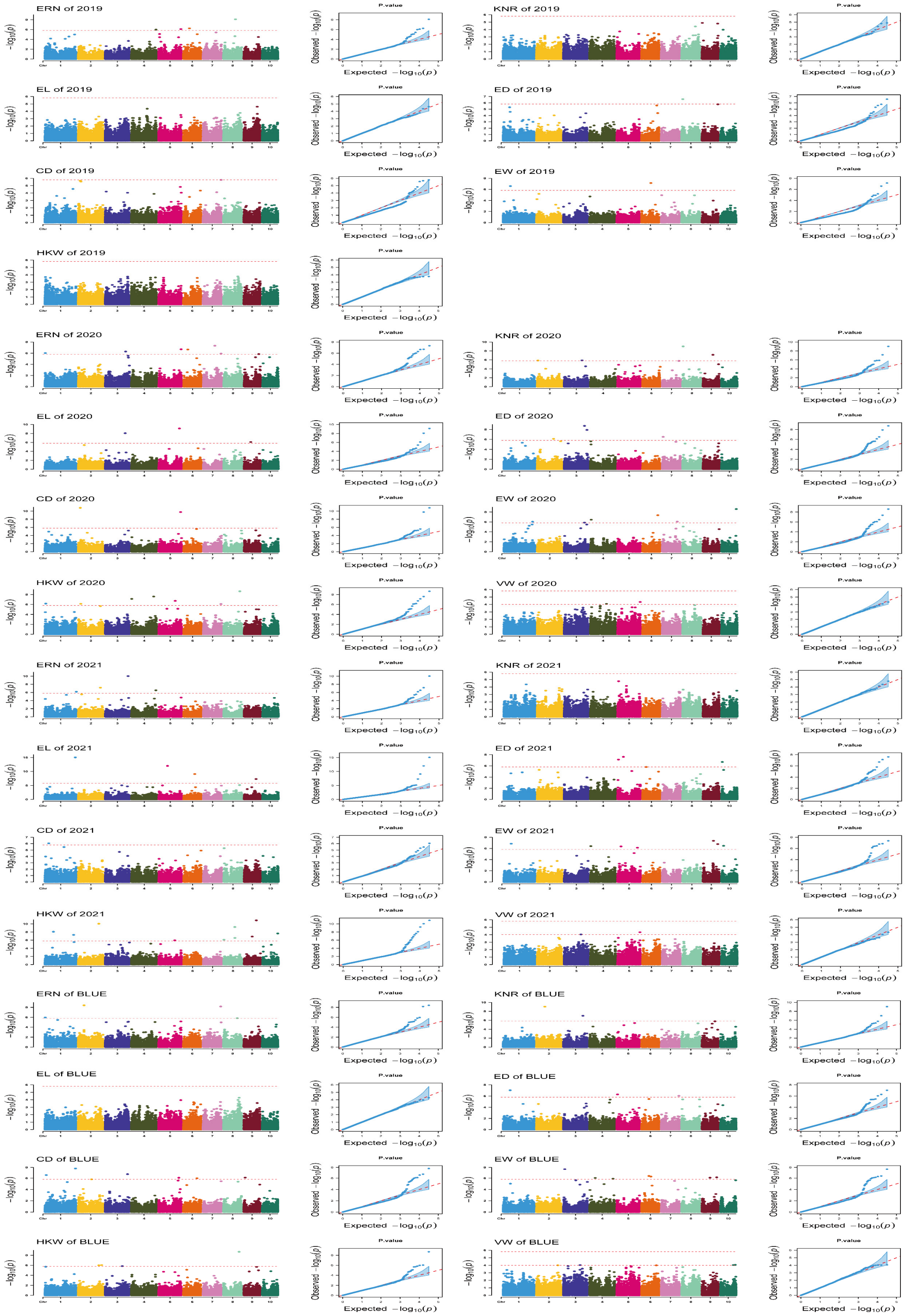

Figure S2. Manhattan map and QQ map of ear traits in different years. ERN, ear row number; KNR, kernel number per row; EL, ear length; ED, ear diameter; CD, cob diameter; EW, ear weight; HKW, hundred kernel weight; VW, volume weight; BLUE, best linear unbiased estimate.

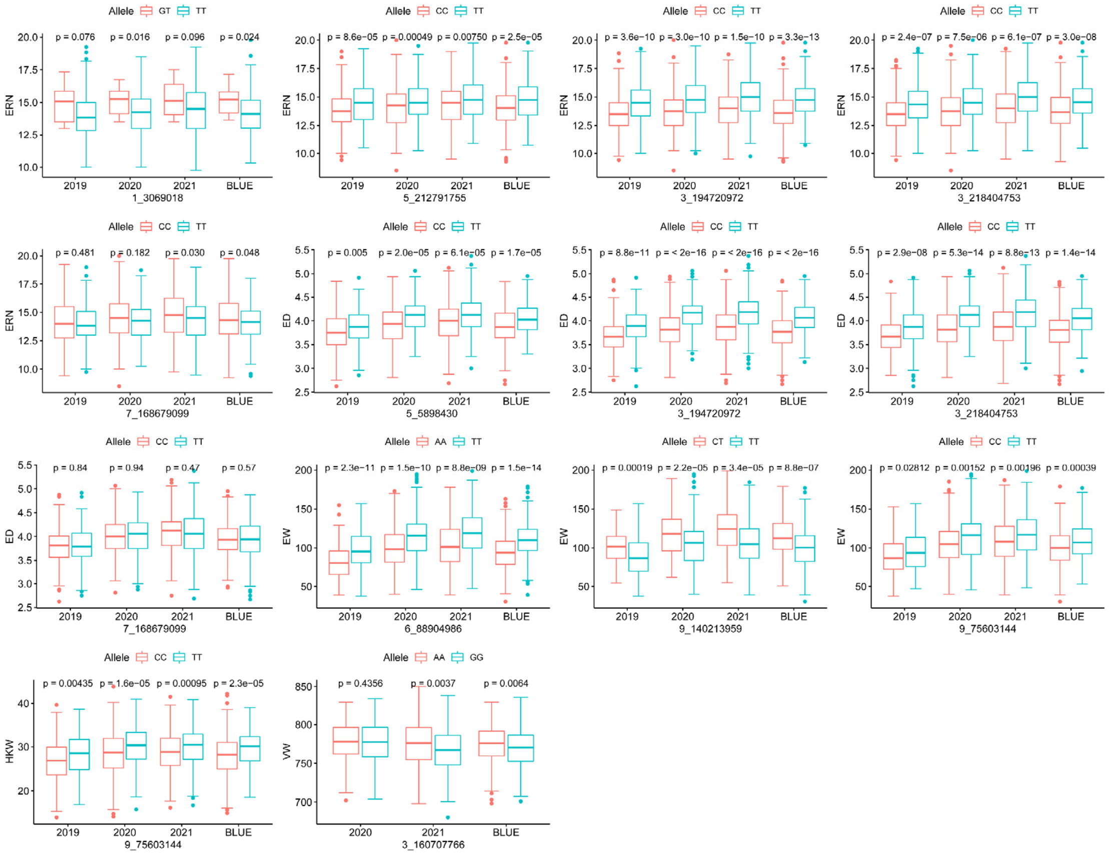

Figure S3. Significance analysis of allele difference of Co-located SNP in different years. ERN, ear row number; ED, ear diameter; EW, ear weight; HKW, hundred kernel weight; VW, volume weight.

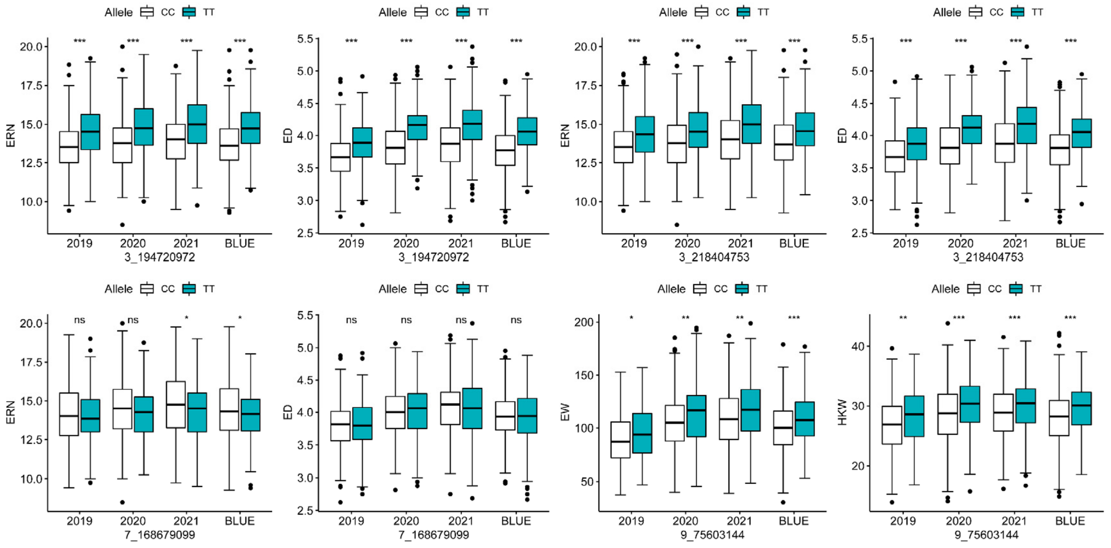

Figure S4. Analysis of allelic effects of colocalized SNPs for ear related traits. ERN, ear row number; ED, ear diameter; EW, ear weight; HKW, hundred kernel weight. *, **and***Significant at $P < 0.05$, P <0.01 and $P < 0.001$, ns indicates no significance.

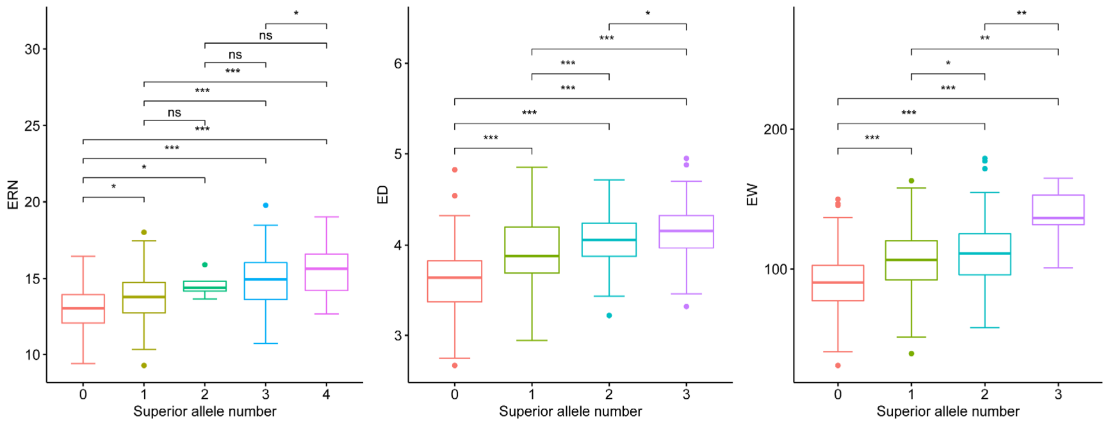

Figure S5. Effect of superior allele number on traits. ERN, ear row number; ED, ear diameter; EW, ear weight. *, **and***Significant at $P < 0.05$, P <0.01 and $P < 0.001$, ns indicates no significance.

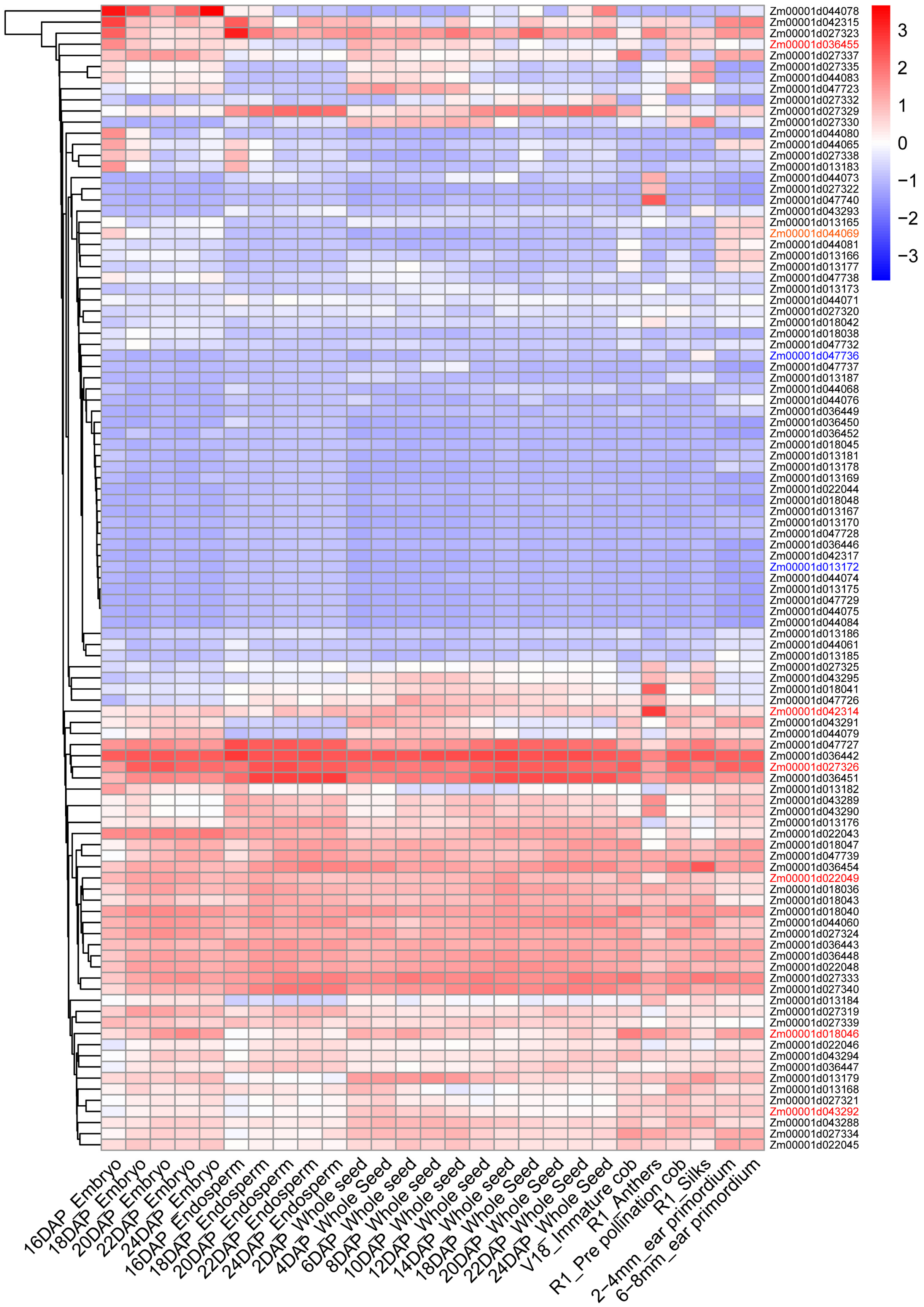

Figure S6. Heatmap of tissue-specific expression patterns of candidate genes. DAP: Days after pollination, V18: Vegetative stage18, R1: Reproductive 1. Standardize data conversion with log_2(FPKM+1).

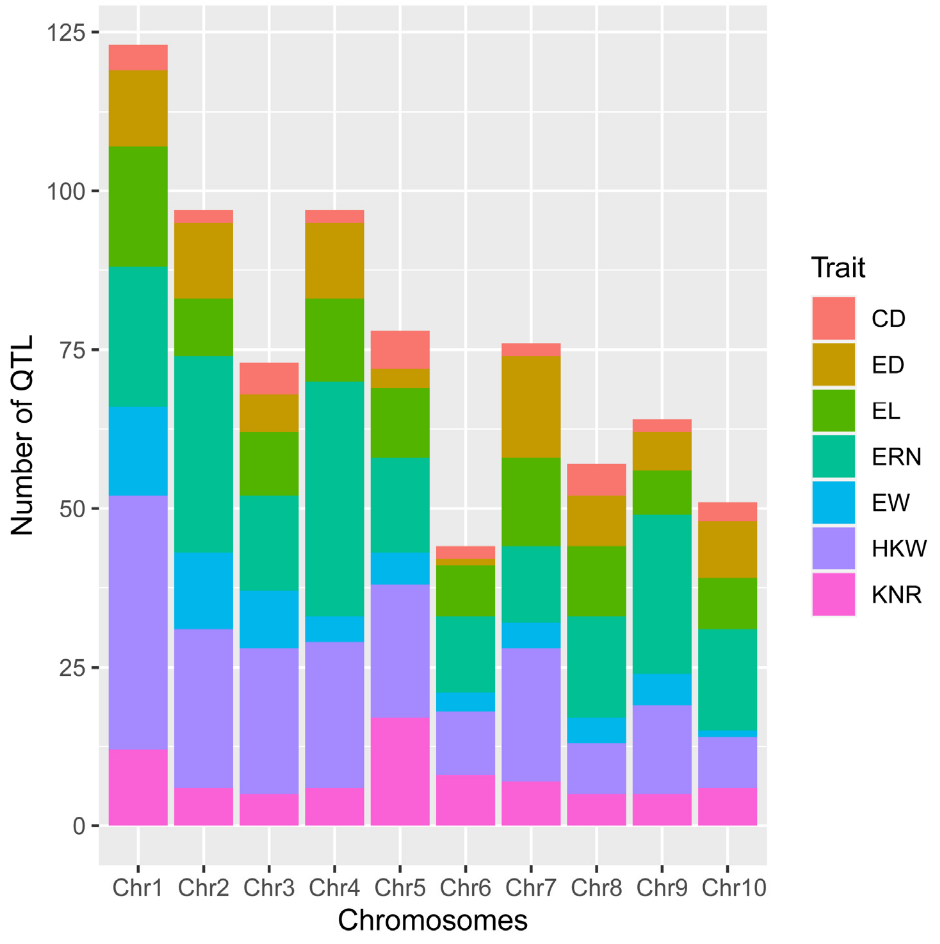

Figure S7. Initial QTL distribution on 10 chromosomes of ear traits. ERN, ear row number; KNR, kernel number per row; EL, ear length; ED, ear diameter; CD, cob diameter; EW, ear weight; HKW, hundred kernel weight; Chr, chromosomes.

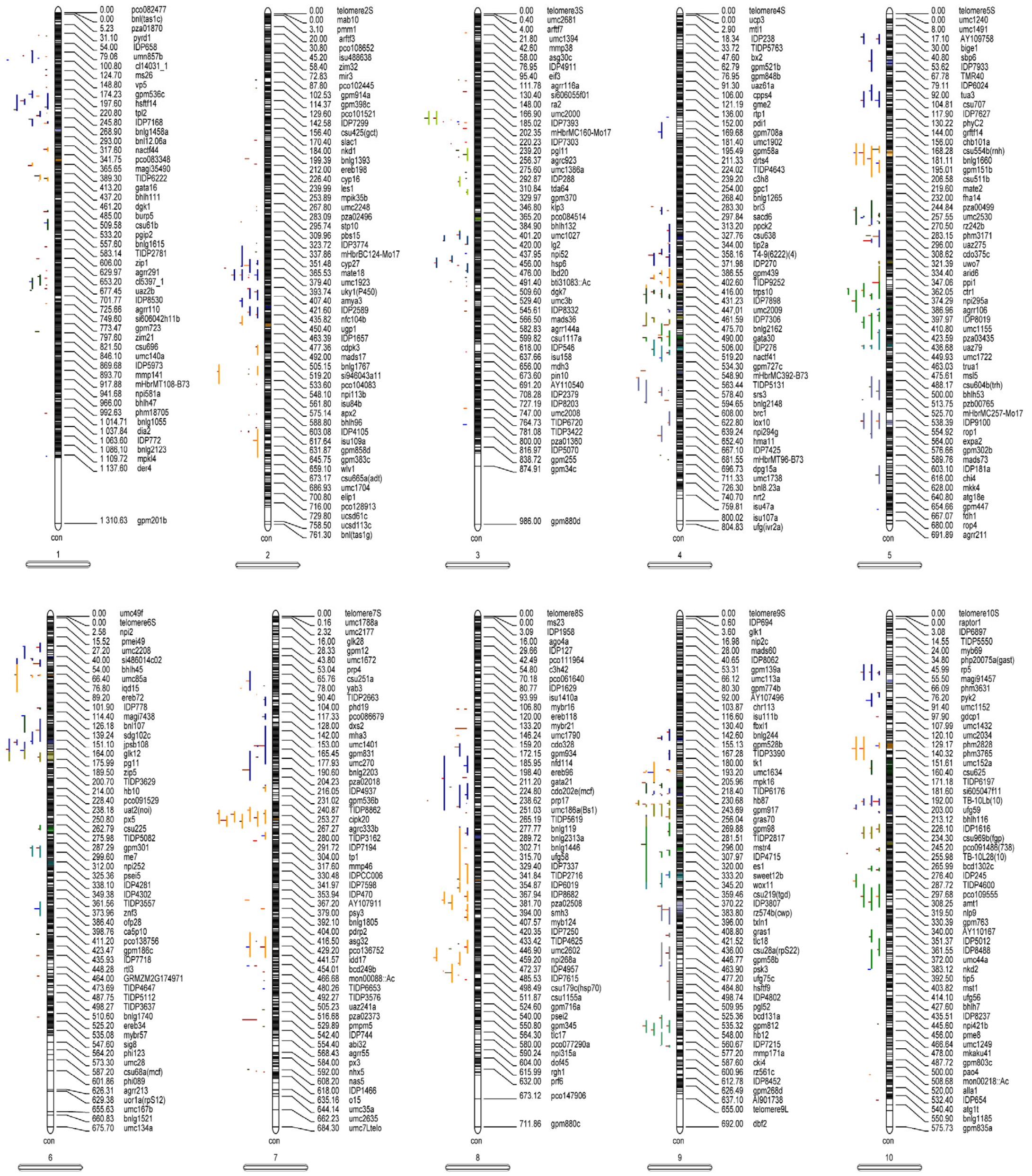

Figure S8. Projection and distribution of QTL and MQTL (Meta QTL) identified for ear traits. Bars on the left side of the chromosome correspond to QTL related to ear traits, black bars within chromosomes represent marker density, colored segments within the chromosome represent MQTL, on the right side of the chromosome are molecular markers and genetic distances (cM).
